# Elective orthognathic surgery: a meta-synthesis of patients’ decision-making experiences

**DOI:** 10.1186/s12903-026-09066-z

**Published:** 2026-07-29

**Authors:** Qing He, Jing Wang, Teng Li, Yayun Wang, Min Liu, Min Zhang, Yongai Zhang

**Affiliations:** 1 School of Nursing and Rehabilitation, Xi’an Medical University, Xi’an, Shaanxi 710021 China; 2 Department of Oral and Maxillofacial Trauma and Orthognathic Surgery, School of Stomatology, The Fourth Military Medical University, Xi’an, Shaanxi 710032 China

**Keywords:** Dentofacial deformity, Orthognathic surgery, Decision making, Qualitative research, Meta-synthesis

## Abstract

**Background:**

Dentofacial deformity is a common and significant oral health issue, which comprehensively affects patients’ quality of life. Orthognathic surgery is a well-established treatment for moderate to severe dentofacial deformities. However, surgical correction is not always elected by patients. Drawing on the Health Belief Model and Shared Decision‑Making as theoretical frameworks, this study systematically synthesized qualitative evidence on patients’ decision-making experiences regarding orthognathic surgery, with the aim of helping them achieve informed preferences.

**Methods:**

A comprehensive search was conducted across PubMed MEDLINE, Ovid MEDLINE, Embase, CINAHL, Scopus, ProQuest, Web of Science, Cochrane Library, SinoMed, CNKI, Wanfang, and VIP, from inception to 2 August 2024. Study screening, quality appraisal, data extraction and synthesis were all conducted by two researchers under the guidance of the JBI manual for evidence synthesis. The GRADE-CERQual approach was used to assess the confidence in each synthesized finding.

**Results:**

A total of 14 studies met the inclusion criteria, including 332 participants alongside 2601 online posts and tweets. All 80 extracted findings were classified into 14 categories and subsequently integrated into 4 synthesized findings: initial intention: inner drivers and self-reconstruction; evaluating pathways: ideal visions versus practical constraints; confirming pathways: role of social networks; decision timing: why now? The confidence in the synthesized findings was moderate, moderate, moderate, and low, respectively.

**Conclusions:**

Patients with dentofacial deformities experience a complex decision-making process that extends beyond clinical factors. This study proposes the Double-Helix Interactive Decision-Making Model, in which patients’ individual beliefs and social contexts interact continuously throughout this process. It also provides a preliminary conceptual framework for patient decision aids. We advocate a shift in clinical practice from technical execution to decision-making empowerment.

**Trial registration:**

PROSPERO CRD42022372729.

**Supplementary Information:**

The online version contains supplementary material available at https://doi.org/10.1186/s12903-026-09066-z.

## Background

Dentofacial deformity (DFD) is a malformation of the jaws due to congenital or acquired factors, or a combination of both. It is characterized by abnormal position and/or size of the maxilla and mandible, and often accompanied by malocclusion [1]. It’s a common and significant oral health condition [1]. DFD impairs essential functions such as chewing [2], swallowing [2], speech [3] and even breathing [4]. It also often leads to concerns about appearance [5]. These concerns can further contribute to low self-esteem [5] and employment difficulties [6], ultimately impacting patients’ quality of life [5, 7–10]. Therefore, early detection and appropriate treatment of DFD are crucial.

As a well-established intervention for moderate to severe DFD, orthognathic surgery is usually performed after jaw growth is complete [1]. For patients with milder symptoms or those who decline surgery, camouflage orthodontics may offer partial correction. However, evidence suggests it is less effective than orthognathic surgery in improving skeletal relationships and optimizing soft tissue profiles [11, 12]. In young patients, orthopedic appliances can be employed to modify jaw development. This approach can help simplify or even avoid the need for future orthognathic surgery [13]. Given their distinct management pathways, such patients are excluded from the present study. Notably, the choice to forgo treatment may also represent a viable treatment option [14].

The surgical correction of DFD presents a decision-making dilemma with significant benefits and considerable burdens. It not only effectively improves the facial appearance and oral function [15], but also has a positive influence on psychosocial health, including confidence, body image, and social adaptability [16], ultimately enhancing patients’ quality of life [7]. However, complications such as bleeding, unexpected fractures, airway obstruction, and peripheral nerve damage may lead to serious consequences [1]. Other notable drawbacks include prolonged recovery periods [17] and high treatment costs [18]. Despite these concerns, studies have shown that even when the significant postoperative complication of neurosensory disturbance is considered, the positive effect of surgery on quality of life remains significant. This suggests that the benefits of undergoing orthognathic surgery may still outweigh its risks [9].

The debate over whether patient motivation for orthognathic surgery is primarily aesthetic [19] or functional [20] has long persisted. Research has indicated that aesthetic concerns are particularly prominent among women and single individuals, while men and married patients often prioritize functional improvements [21]. Moreover, some scholars contend that psychological needs of patients may be more intense than other motivations [22]. Overall, improvements in aesthetics, function, and confidence constitute the three primary motivations for surgical intervention [23]. Besides intrinsic factors, external influences such as clinician referrals and recommendations from family members or friends also contribute to the decision to seek surgical treatment [24]. However, patients also express the wish to avoid potential complications [25]. Notably, financial considerations do not appear to be a major barrier, as patients often proceed with surgery once they are able to afford it [26]. Although Class II malocclusion is more prevalent, patients with Class III malocclusion opt for surgical correction more frequently. This may be explained by the fact that Class II malocclusion is easier to correct with camouflage orthodontics [25].

Overall, postoperative patient satisfaction is generally high and closely associated with their preoperative motivations [25, 27, 28]. The greater the emphasis on aesthetic changes or functional improvements, the higher the satisfaction with treatment outcomes [27]. Importantly, it is crucial to identify whether patients’ psychological needs have been met. Otherwise, even with optimal surgical results, dissatisfaction may arise [10]. Moreover, persistent complications [21, 25, 28], prolonged treatment durations [28], and high surgical costs [24] also influence patient satisfaction.

Much existing research has quantified patients’ motivations before orthognathic surgery [21, 23, 25, 27] and their perceptions after the procedure [21, 25, 27, 28]. These studies outline the key structural elements at the start and end of the decision-making pathway. However, patients’ psychological experiences and behavioral logic throughout the decision-making process remain poorly understood. A clear understanding of this process can help identify factors that facilitate or impede their treatment choices. In turn, this can support patients in navigating the process smoothly. Although qualitative research on patients’ decision-making experiences regarding orthognathic surgery has increased in recent years [29–32], such studies adopt diverse perspectives, leading to fragmented findings and general recommendations. To our knowledge, higher-level syntheses of qualitative evidence have not yet been published.

The Health Belief Model (HBM) and Shared Decision-Making (SDM) provide well-established theoretical frameworks for understanding patients’ decision-making process. The HBM aims to explain and predict why individuals adopt or avoid specific health behaviors. According to this model, patients are most likely to act when they perceive significant threats and believe that the benefits of the action outweigh its barriers [33]. SDM emphasizes collaborative decision-making between clinicians and patients based on shared information [34]. Recently, scholars have noted that the influence of family and society is gaining increasing recognition within SDM [35]. The contextualized SDM that accounts for patients’ social networks may represent an important evolution of the theory. These theories suggest that patients’ decision-making is a complex process. However, how these mechanisms manifest in the context of orthognathic surgery warrants focused exploration.

In summary, this study adopted the HBM and SDM as fundamental frameworks. A meta-synthesis was performed to investigate the decision-making experiences of patients with DFD regarding orthognathic surgery, thereby offering guidance to help patients achieve informed preferences. This work may provide a basis for the development and implementation of a clinical decision support system for orthognathic surgery. The protocol for this study has been registered with PROSPERO (CRD42022372729).

## Methods

### Study design

This meta-synthesis integrated qualitative evidence on the decision-making experiences of patients with DFD regarding orthognathic surgery. The study was conducted following the Joanna Briggs Institute (JBI) manual for evidence synthesis [36] and adhered to the Enhancing transparency in reporting the synthesis of qualitative research (ENTREQ) statement [37].

### Inclusion and exclusion criteria

Studies were included if they met the criteria for participants, phenomena of interest, context, and types of studies shown in Table 1. In addition, studies that were duplicated, unavailable full-text, or not in Chinese or English were excluded.

**Table 1:** Inclusion and exclusion criteria

Criteria	Inclusion	Exclusion
Participants	Patients with DFD eligible for orthognathic surgery	Patients under 16 (due to ongoing jaw growth and parental influence on decision-making)
Phenomena of interest	Patients’ experiences of considering or reflecting on the decision‑making process for orthognathic surgery	
Context	Any healthcare institution or other appropriate setting	
Types of studies	Qualitative research and mixed-methods studies utilizing qualitative approaches. For mixed-methods studies, only the qualitative data were extracted	Quantitative research or mixed-methods studies where qualitative data cannot be separated

### Search strategy

We searched PubMed MEDLINE, Ovid MEDLINE, Embase, CINAHL, Scopus, ProQuest, Web of Science, Cochrane Library, SinoMed, CNKI, Wanfang, and VIP, from their inception to 2 August 2024. A systematic search for grey literature was not conducted. However, any relevant grey literature identified within the default search scope of the core databases was considered for inclusion. The literature search combined subject headings and free-text terms. The complete search strategy for all databases is detailed in Appendix A of the Supplementary Material.

### Study screening

Duplicate records were identified using EndNote X9 and manual checking. Subsequently, studies were screened based on inclusion and exclusion criteria. Titles and abstracts were screened first, and full texts of potentially eligible studies were obtained for detailed evaluation. Two researchers independently performed the screening and documented reasons for exclusion. Any disagreements were resolved through discussion.

### Assessment of methodological quality

The methodological quality of qualitative studies was assessed using the JBI Critical Appraisal Checklist for Qualitative Research. This tool consists of 10 items rated as “yes,” “no,” “unclear,” or “not applicable.” Each study was ultimately assigned to “include,” “exclude,” or “seek further info.” Two researchers independently assessed methodological quality. Any differences were discussed with a third reviewer to reach consensus.

### Data extraction

Two researchers independently extracted key information from the included studies using a modified JBI Qualitative Data Extraction Tool, including author information, publication year, country, participants, phenomena of interest, context, methodology, data collection, data analysis, and findings. Each finding extracted was accompanied by an illustration and assigned a level of credibility rated as “unequivocal” (clearly supported by data and not open to challenge), “credible” (supported by data but open to challenge), or “not supported” (unsupported by data). Findings, along with their illustrations and credibility levels, are detailed in Appendix B of the Supplementary Material.

### Data synthesis

The meta-aggregative approach of JBI was employed to synthesize the qualitative findings with established credibility. Following the JBI manual for evidence synthesis, both “unequivocal” and “credible” findings were included in the aggregation. Findings rated as “not supported” were excluded from the process [36]. The coding process was facilitated using NVivo 15. The findings and their illustrations were identified through line-by-line reading of the included studies. Based on similarity in meaning, findings were categorized to form categories. Subsequent findings were continuously compared with existing categories and new categories could be generated to accommodate emerging themes. Finally, these categories were synthesized to produce synthesized findings. Two researchers independently conducted the meta-aggregation. Any discrepancies were discussed with a third reviewer to reach consensus.

### Assessment of confidence in review findings

The Confidence in the Evidence from Reviews of Qualitative research (GRADE-CERQual) approach was used to assess confidence in review findings [38]. This assessment considers four components: methodological limitations, relevance, coherence, and adequacy of data. There are four levels of confidence: high, moderate, low, or very low. All review findings are initially rated as “high confidence” and then downgraded based on concerns in any of the four components. Two researchers independently assessed confidence. A third reviewer was consulted when necessary.

### Reflexivity statement

The research team comprised seven members. Four members (QH, JW, TL, YZ) specialized in qualitative evidence synthesis, while three members (YW, ML, MZ) possessed direct clinical experience in orthognathic surgery. None had ever undergone orthognathic surgery. Prior to data synthesis, we assumed that functional impairment and appearance concerns were the main drivers of surgical decision-making. During the synthesis process, we found patients’ decision-making was more complex than anticipated. Through regular team discussions, we revisited our initial assumptions and clarified the role of additional factors. Team members with clinical experience learned about evidence synthesis, while those with methodology expertise learned about orthognathic surgery. This mutual learning shaped our final interpretations. To mitigate potential bias arising from the clinical background of team members, non-clinical members led the initial synthesis, while clinical members refined the review findings.

### Robustness checks and cross-contextual comparisons

#### Robustness checks

To assess the robustness of the review findings, we sequentially excluded the following groups of studies and re-synthesized the data to observe whether the 4 synthesized findings and 14 categories remained unchanged:

- a) Studies exclusively using social media data.
- b) Eligible grey literature identified within core databases.
- c) Studies with the lowest-quality JBI scores.
- d) Studies focusing on surgical experience rather than surgical decision-making.

#### Cross-contextual comparisons

To explore the applicability of the review findings across different contexts, we compared the distribution of the 4 synthesized findings and 14 categories between the following groups of studies:

- a) By healthcare system: Given that most studies were conducted in the UK, where the National Health Service (NHS) has unique characteristics in terms of free healthcare, structured referral, and waiting time, we compared the NHS group with the non-NHS group.
- b) By treatment outcome: In some studies, all patients had decided to undergo or had already undergone surgery. However, several other studies included not only patients who underwent surgery but also some who declined surgery or were undecided, and data from these subgroups could not be extracted separately. Therefore, we compared the surgery-only group with the mixed group (patients who underwent surgery, declined surgery, or were undecided).

## Results

### Study inclusion

**Fig. 1:**
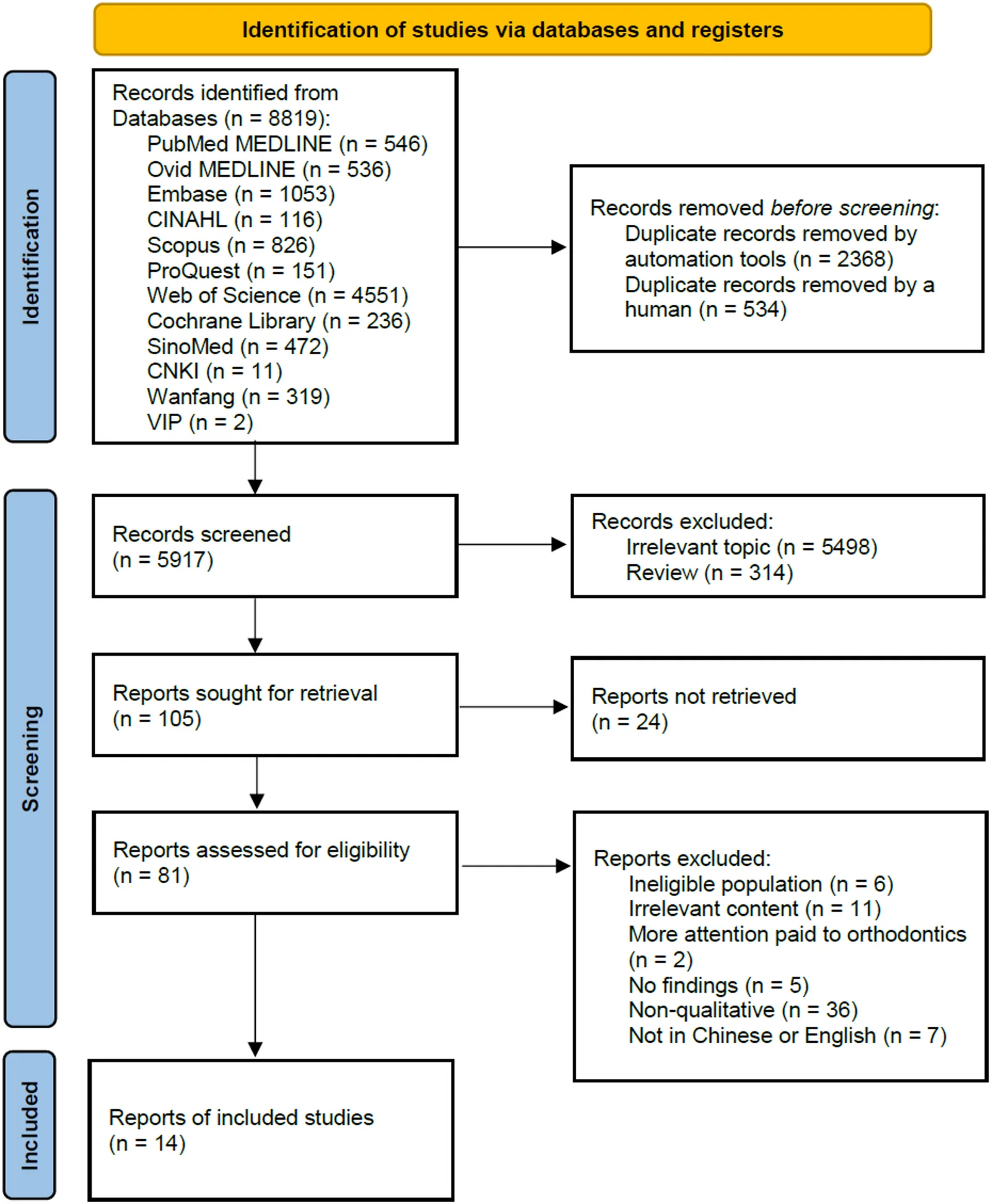
PRISMA flow diagram

A total of 8819 records were initially retrieved from the databases. After removing 2902 duplicates through both automated and manual processes, 105 records were identified through title and abstract screening. Following the exclusion of 24 records without full texts, 14 studies ultimately remained through full-text review. The PRISMA flow diagram is presented in Fig. 1.

### Methodological quality

All studies scored “yes” on more than 7 items, which indicated acceptable methodological quality. In order to fully utilize the available data, we did not exclude any studies. The methodological quality assessment of included studies is shown in Table 2.

**Table 2:** Methodological quality assessment of included studies

Study	Q1	Q2	Q3	Q4	Q5	Q6	Q7	Q8	Q9	Q10
Hu et al. (2019) [29]	Y	Y	Y	N	Y	N	N	Y	Y	Y
Ryan et al. (2012) [30]	Y	Y	Y	Y	Y	Y	Y	Y	Y	Y
Ryan et al. (2012) [39]	Y	Y	Y	Y	Y	Y	Y	Y	Y	Y
Stirling et al. (2007) [40]	Y	Y	Y	Y	Y	N	N	Y	Y	Y
Paul et al. (2022) [31]	Y	Y	Y	Y	Y	Y	Y	Y	Y	Y
Broder et al. (2000) [41]	Y	Y	Y	Y	Y	N	N	Y	Y	Y
Ansari et al. (2024) [42]	Y	Y	N	Y	Y	N	N	Y	Y	Y
Antov (2023) [32]	Y	Y	Y	Y	Y	Y	Y	Y	Y	Y
Travess et al. (2004) [43]	Y	Y	Y	Y	Y	N	Y	Y	Y	Y
Bhamrah et al. (2015) [44]	Y	Y	Y	Y	Y	N	N	Y	Y	Y
Watts et al. (2018) [45]	Y	Y	Y	Y	Y	N	Y	Y	Y	Y
Kettle et al. (2021) [46]	Y	Y	Y	Y	Y	N	Y	Y	Y	Y
Cadogan and Bennun (2011) [47]	Y	Y	Y	Y	Y	N	N	Y	Y	Y
Liddle et al. (2018) [48]	Y	Y	Y	Y	Y	N	Y	Y	Y	Y

*Y* Yes, *N* No, *U* Unclear, *N/A* Not applicable

Q1. Congruity between the stated philosophical perspective and the research methodology

Q2. Congruity between the research methodology and the research question or objectives

Q3. Congruity between the research methodology and the methods used to collect data

Q4. Congruity between the research methodology and the representation and analysis of data

Q5. There is congruence between the research methodology and the interpretation of results

Q6. Locating the researcher culturally or theoretically

Q7. Influence of the researcher on the research, and vice-versa, is addressed

Q8. Representation of participants and their voices

Q9. Ethical approval by an appropriate body

Q10. Relationship of conclusions to analysis, or interpretation of the data

### Characteristics of included studies

A total of 14 studies were included in this review. The majority of studies were conducted in the UK (*n* = 9), the remaining studies were from the US (*n* = 2), China (*n* = 1), Australia (*n* = 1), and Switzerland (*n* = 1). The methodology encompassed descriptive qualitative studies (*n* = 5), exploratory qualitative studies (*n* = 2), phenomenological studies (*n* = 3), grounded theory study (*n* = 1), narrative study (*n* = 1), and mixed‑methods studies (*n* = 2). In terms of sampling, purposive sampling was most common (*n* = 10), followed by consecutive sampling (*n* = 2). The two studies using social media data respectively applied random sampling and consecutive sampling. Across all included studies, the total dataset comprised 332 participants alongside 2601 online posts and tweets. Most studies (*n* = 9) were conducted in settings such as hospitals, schools, or other undisturbed locations, while two studies used telephone or remote conferencing. The remaining three studies did not specify the context. Data collection primarily involved semi‑structured interviews or focus group discussions (*n* = 11). Additionally, one study employed structured interviews, while the remaining two studies utilized anonymized public data. Data analysis methods varied according to the methodology and included content analysis, thematic analysis, interpretative phenomenological analysis, and open and selective coding. The characteristics of included studies are presented in Table 3.

**Table 3:** Characteristics of included studies

Author/Year/Country	Sampling/Participants	Phenomena of interest	Context	Methodology	Data collection	Data analysis
Hu et al. (2019) [29] China	Purposive sampling/Patients consulted in the Department of Oral and Craniomaxillofacial Surgery, Center of Craniofacial Orthodontics in Shanghai Ninth People’s Hospital (*n* = 10)	The experience associated with patient’s decision to have or not have orthognathic treatment	A place that is convenient for conversation and free from distractions	Descriptive qualitative study	Semi-structured, face to face in-depth interviews	Colaizzi content analysis
Ryan et al. (2012) [30]* UK	Purposive sampling/Patients with a dentofacial deformity aged 16 years and over, who had been accepted for orthognathic treatment, but had not yet commenced treatment (*n* = 18)	Impact of dentofacial deformity and motivation for orthognathic treatment	Non-clinical environment	Exploratory qualitative study	Semistructured in-depth interviews	Critical Qualitative Theory (CQT), a type of thematic analysis
Ryan et al. (2012) [39]* UK	Purposive sampling/Patients with a dentofacial deformity aged 16 years and over, who had been accepted for orthognathic treatment, but had not yet commenced treatment (*n* = 18)	Orthognathic patients’ expectations of the outcome of orthognathic treatment	Non-clinical environment	Exploratory qualitative study	Semistructured in-depth interviews	Critical Qualitative Theory (CQT), a type of thematic analysis
Stirling et al. (2007) [40] UK	Purposive sampling/Patients over 16 years old diagnosed with skeletal anomalies were making (or had previously made) choices about orthognathic treatment (*n* = 59)	The process of decision making by orthognathic treatment patients in more detail	By telephone	Mixed-methods study	Semi-structured telephone interviews	Thematic content analysis
Paul et al. (2022) [31] UK	Purposive sampling and theoretical sampling/Patients 6 to 8 weeks postsurgery, in the decision-making stage or in long-term follow-up (*n* = 22), supplemented by online blogs and forums on jaw surgery (number not reported)	Patients’ experience of decision making for orthognathic treatment	A closed room within the dental hospital or in the adjoining dental school	Grounded theory study	Face-to-face interviews and online blogs and forums	Open and selective coding
Broder et al. (2000) [41] US	Purposive sampling/Patients who were evaluated within the past 18 months and had not yet undergone orthognathic surgery (*n* = 118)	Issues related to consumer decision making	University of Medicine and Dentistry of New Jersey and University of North Carolina	Descriptive qualitative study	Focus groups and semi-structured individual interviews	Thematic analysis
Ansari et al. (2024) [42] Australia	Consecutive sampling/Patients undergoing orthognathic surgery by a tertiary oral and maxillofacial surgeon (*n* = 18)	Patient motivations and expectations of orthognathic surgery	Comfortable workplace interview rooms	Descriptive qualitative study	Structured interviews	Thematic analysis
Antov (2023) [32] UK	Consecutive sampling/Patients aged 16 or over, suitable and willing to undergo orthognathic surgery (*n* = 14)	The psychology of orthognathic patients	Use Microsoft Teams remotely	Phenomenological study	Semi-structured interviews	Interpretative phenomenological analysis
Travess et al. (2004) [43] UK	Purposive sampling/Patients who had received combined orthodontic and orthognathic treatment (*n* = 26)	Patients’ views of combined orthodontic orthognathic treatment	A non-clinical environment outside the hospital	Mixed-methods study	Focus group discussions	Thematic analysis
Bhamrah et al. (2015) [44] US	Posts selected from the online orthognathic discussion forum by a random number generator (*n* = 1912)	Information that orthognathic patients share and discuss with peers away from the clinical environment	Not mentioned	Descriptive qualitative study	Search for an orthognathic discussion forum with the terms “discussion forum,” “orthognathic surgery,” “surgical orthodontics,” and “jaw surgery”	Thematic analysis
Watts et al. (2018) [45] Switzerland	A sample of cross-sectional consecutive tweets posted on Twitter relating to orthognathic surgery (*n* = 689)	Patients’ thoughts and experiences surrounding orthognathic surgical treatment	Not mentioned	Descriptive qualitative study	Search for the keywords “jaw surgery” or “#jawsurgery” to identify relevant tweets	Thematic analysis
Kettle et al. (2021) [46] UK	Purposive sampling/Patients aged 16 and over who had undergone orthognathic surgery for the correction of jaw asymmetry within the last five years (*n* = 15)	Patients’ experiences of orthognathic treatment for facial asymmetry	Two UK sites	Narrative study	In-depth, face-to-face, qualitative interview	Thematic analysis
Cadogan and Bennun (2011) [47] UK	Purposive sampling/Patients aged 20–25 years had had operations between 2 and 4 years previously (*n* = 7)	Experiences of participants with craniofacial conditions before, during, and after orthognathic surgery	Not mentioned	Phenomenological study	Semi-structured interviews	Interpretative phenomenological analysis
Liddle et al. (2018) [48] UK	Purposive sampling/Patients aged between 18 and 25 years and who had undergone a bimaxillary osteotomy (*n* = 7)	Experiential account of the processes of change associated specifically with orthognathic surgery	An NHS teaching hospital	Phenomenological study	Semi-structured interviews	Interpretative phenomenological analysis

*These two studies shared overlapping samples (*n* = 18)

### Review findings

A total of 80 findings were extracted from the 14 included studies. Among them, 75 findings were rated as “unequivocal,” while the remaining 5 findings were rated as “credible.” All findings were included in the subsequent meta-aggregation. These findings were classified into 14 categories and then integrated into 4 synthesized findings. Findings, categories, and synthesized findings are detailed in Appendix C of the Supplementary Material.

#### Synthesized finding 1: initial intention: inner drivers and self-reconstruction

##### Category 1: functional and aesthetic needs

The functional impacts of DFD primarily manifested in areas such as mastication, speech, sleep, and pain. Patients reported difficulty biting normally [39] and chewing food thoroughly [29]. The consumption of large pieces of food, such as steak [41, 43] or sandwiches [45], required excessive chewing and posed considerable difficulty. This led to distress over being unable to enjoy meals [30, 44]. Speech was unclear [31, 41, 42] or muffled [42]. In cases where the lower lip was pressed by the upper teeth, patients needed to force the jaw forward during speech [44]. Furthermore, sleep was often severely disrupted by symptoms including airway obstruction [44], breathing difficulties [44], and snoring [29]. Physical discomforts such as headaches [46] and jaw pain [46] were also reported, with exacerbation during yawning or eating [42]. The condition itself was further associated with potential problems like temporomandibular joint inflammation [29]. For these reasons, patients considered orthognathic surgery to restore essential functions such as normal eating, speaking, and breathing [42].

*“I get to speak properly*,* breathe properly*,* eat properly*,* be a healthier person.”* [42] *(p. 49*,* P8)*

Patients emphasized their distinct facial features such as overjet [42], underbite [29], crooked teeth [42], lip protrusion [29], long jaw [44], facial asymmetry [29], and unusual expressions [30]. They perceived themselves as comical and ugly [29, 40], describing their appearance as Peking Man [29], a witch [32], an ugly duckling [47], or even a bulldog [32]. Some also recognized that DFD could contribute to premature ageing [40]. Patients generally disliked their own appearance [43] and even felt disgusted [46]. They either avoided laughing or covered their mouths when laughing [29, 44, 48], while they either refused to be photographed or lowered their heads when photographed [29, 32, 46, 48]. Patients expressed a strong desire for a normal appearance, including aligned teeth [31, 45], a naturally closed mouth [39], a normal chin [31, 32, 39], and a harmonious facial profile [32, 42]. They hoped to be able to open their mouth and show their teeth, so that they could laugh openly [31, 40, 44], be photographed bravely [32], and feel just like any ordinary person [29, 31, 40, 47]. Notably, influenced by screen aesthetics, a minority of patients aspired to a model-like appearance [47]. These aesthetic needs could arise during adolescence when self-consciousness peaked [40, 47], or later in life as expectations regarding facial appearance increased [29].

*“Umm … just to look normal. To … have a normal smile and a nice smile … a normal chin*,* a normal bottom jaw … normal as anyone.”* [31] *(p. 262*,* Marie)*

##### Category 2: psychosocial needs

Functional and aesthetic concerns jointly placed patients in a predicament of social interaction. Teasing, ridicule, and bullying from peers and even siblings during childhood led to persistent psychological trauma in patients [30, 32, 40, 48]. In adulthood, they might face malicious judgment due to their eating habits [46] or distinctive appearance [30, 47]. Unabashed stares from strangers triggered an urge to shrink [29, 32]. Patients’ self-protection and defensive mindset were prominent [30]. This manifested as social avoidance [30, 32], reluctance to go out [46], and difficulty establishing intimate relationships [30]. Patients’ careers were also significantly compromised [30, 48]. Despite these barriers, patients still hoped to step out, fit in, and transform their lives [30, 32, 39, 41, 42, 48].

*“I don’t like where I’m going at the moment*,* and that’s why I’m doing something about it*,* because I want my life to change*,* because I want to be out there*,* I want to be with the outside crowd*,* I want to go out*,* I want to talk to people*,* I want to be friendly with my colleagues and everyone.”* [30] *(p. 739*,* P7)*

Lack of confidence [29–32, 42] was the most frequently mentioned psychological experience, manifesting as introversion [29], inferiority [29], loneliness [29], emotionalism [30], frustration [30, 40, 42, 46], and even depression [30]. It further fostered negative body image [29, 30, 42] and an unjust sense of victimization [30]. Patients’ expectations for treatment focused on feeling relaxed, lowering defensiveness, and achieving self-reconciliation. They regarded these as essential for attaining happiness [39, 42].

*“Just being able to relax and forget about your appearance*,* it’s me being happy with my appearance and saying okay*,* that’s all okay now*,* I don’t have to think about that*,* and I’ll just get on with everything and be like everybody else*,* because I’ve seen what everybody else is like.”* [39] *(p. 2651)*

##### Category 3: self-schema

When confronted with social challenges, patients exhibited two primary coping strategies. One was avoidant coping, characterized by passively withdrawing to distance themselves from distressing situations [30]. These patients remained quiet to avoid attention [32]. The other was compensatory coping, involving actively disguising themselves to manage others’ perceptions [30]. These patients acted outgoing to divert attention [32]. Some patients achieved positive self-reconstruction following psychological distress, demonstrating a strong self-identity despite prejudice [47].

*“I still do get called the odd name by the odd person*,* but the difference with me now is I’ve built up enough confidence and strength in myself to say*,* ‘that’s your opinion*,* if you think that*,* fine*,* but I don’t care to listen to it’.”* [47] *(p. 378)*

Regarding expectations of orthognathic surgery, patients could be categorized into four distinct types [39]. “Metamorphosizers” sought both physical and nonphysical renewal, aiming not only for a perfect appearance but also for a complete transformation in their lives. “Pragmatists” focused on specific physical improvements. They did not expect to alter their overall life through treatment, viewing their concerns primarily as dentofacial issues. “Shedders” pursued external changes to alleviate internal burdens. They felt satisfied if their lives improved, even with limited physical alteration. “Evolvers” preferred cautious progress, with relatively low expectations for both physical and nonphysical changes. Their motivation for treatment often came from significant others. Additionally, confidence in one’s own decision‑making ability was a key internal factor driving patients from hesitation to action [40].

*“Feel I made the right decision for where I am in life.”* [40] *(p. 119*,* P10)*

##### Category 4: alternative treatments

Patients perceived that orthognathic surgery was the only viable treatment option [29, 40, 48]. They believed that orthodontic treatment alone did not provide permanent results, as removing the braces could lead to relapse [40]. Moreover, since the underlying skeletal issues remained unaddressed, they still considered their appearance abnormal [29, 40].

*“They said if I took the brace off it would go back to before.” “They said it would make my nose look huge as my bottom jaw is too small.”* [40] *(p. 120*,* P18 & P8)*

#### Synthesized finding 2: evaluating pathways: ideal visions versus practical constraints

##### Category 5: surgical outcomes

Patients looked forward to a dual transformation of body and psychology from the surgery, but they also felt deeply anxious about the results. They worried that the change might be insufficient, resulting in minimal or even no improvement compared to their preoperative appearance [29, 40–42]. At the same time, they feared that the alteration could be excessive, making adaptation difficult [40–42]. Their deeper concern, however, was that even if their looks turned out as hoped, the psychological distress still persisted [40]. The core of this anxiety stemmed from the irreversibility of the surgery, which could lead to a lifetime of self-scrutiny [48].

*Concern about the … extent of facial change (“too much” or “why bother”).* [41] *(p. 255)*

*“Worried that I will look better after surgery but I’ll still feel the same.”* [40] *(p. 119*,* P100)*

##### Category 6: fear of treatment

Specific risks associated with orthognathic surgery, such as anesthetic incidents [29, 40], drug allergies [42], bleeding [42], jaw fractures [40], muscle dysfunction [32], nerve damage [32, 42], facial sensory disturbance [41], infection [32], swelling [41], and pain [29, 32, 41, 42], posed considerable barriers to decision-making. Although extremely rare, severe complications including blindness [32] and death [32] necessitated heightened caution. Moreover, long-term potential risks, such as unpredictable soft tissue changes [29, 32] and late relapse [29], remained concerns for patients. Patients also needed to prepare for general burdens, including hospital-related anxiety [31] and short-term postoperative weight loss [31]. Notably, some patients viewed undergoing surgery at an authoritative medical center as a key strategy for risk-management [29].

*“If the surgery is performed at a major hospital renowned for orthognathic surgery*,* the surgical risks should not be too high.”* [29] *(p. 515*,* A5)*

##### Category 7: treatment duration

Orthognathic surgery entailed a long treatment duration [32, 40, 41] and was difficult to interrupt [40], which forced patients to put their lives on hold [42]. Consequently, patients typically delayed seeking treatment until their personal affairs were in order and their living conditions were relatively stable [31, 41].

*“Long-winded process because once started hard to stop.”* [40] *(p. 120*,* P12)*

##### Category 8: financial considerations

Orthognathic surgery was costly and had limited insurance coverage in some cases, which constituted a significant concern for patients [29, 41, 45]. Most patients relied on family support [29, 41, 45] or long-term savings [29] to afford the procedure, and actively chose more affordable institutions for the surgery [41]. Meanwhile, some patients did not perceive the cost as a constraint [29].

*“Maybe I am just stuck like this for life. What can I do? I am broke and ugly. I feel like a loser every day.”* [29] *(p. 515*,* A6)*

##### Category 9: service experience

The overall service experience influenced patients’ decision-making. Cleanliness of the clinical environment was fundamental to establishing trust in healthcare [41], while ostentatious equipment was not considered a practical choice for patients [41]. Patients also expressed a need for emotional support from mental health professionals [32].

*“I think it’s a great idea. Its someone else to talk to*,* a professional who would understand*,* who would know what to say.”* [32] *(p. 5*,* P4)*

#### Synthesized finding 3: confirming pathways: role of social networks

##### Category 10: information support

Among various information sources, clinicians were the most common and trusted [29–32, 40–42, 46, 48], while patients with similar experiences were the most preferred [29, 31, 32, 46]. Other sources, such as family members and friends [30, 46], also influenced patients’ decision-making.

Information was presented mainly through oral explanations, written materials, and visual simulations. Patients reported that the provision of information relied predominantly on verbal communication, with other formats being inadequate [31]. Evaluations of written materials, such as pamphlets, were mixed. Both the comprehensiveness of the information provided [32, 40] and its alignment with patients’ needs [31] shaped such views. Nevertheless, some patients believed that these materials could stimulate reflection and prompt further medical consultations [40]. In contrast, patients generally expressed positive views on visual simulations [29, 41, 48].

The core content provided by clinicians was treatment plans [41]. Orthodontics before and after surgery [29, 41], treatment duration [29, 46], and surgical procedure [29, 42] were all key treatment details that patients expected to obtain. Various treatment options should also be provided [30, 31]. Furthermore, helping patients recognize their own dentofacial discrepancies was a priority for the initial consultation, while these issues had been downplayed out of kindness by family members or friends [46]. Such guidance could assist patients in confronting their differences [32, 46], but it might also intensify their concerns about defects or even lead to an obsession [30, 40, 42, 48]. Some patients described the information from their clinicians as clear and definitive [31, 46]. However, several patients complained that the information they received about the treatment plan was insufficient [40] and largely focused on postoperative appearance changes [40]. Additionally, the lack of timely [40, 48] and proactive [31, 48] communication often left patients psychologically unprepared. Most patients expressed a desire for more comprehensive information [29, 31]. Interestingly, some patients noted that excessively detailed surgical information could be frightening and even lead to treatment abandonment. They simply wanted to complete the surgery [31, 40]. Other reported problems involved excessive use of medical terminology [40] and no clear recommendations from clinicians regarding whether to undergo surgery [40].

*“I just do not trust information from others. I need to hear it directly from my doctor or see the official notice. Only then can I decide.”* [29] *(p. 516*,* A9)*

##### Category 11: social influence

Social roles had a significant influence on patients’ decision-making. Parents were the most frequently mentioned, whose opinions on whether to undergo surgery were authoritative [40]. Many parents insisted that surgery was unnecessary [29, 32, 46], and even believed that the decision should be made based on the partner’s opinion after marriage [29]. Some parents supported surgery when safety could be assured [29, 48]. Patients also cited avoiding embarrassment for their child as a reason for choosing surgery [40]. Family members with the same condition encouraged patients to undergo surgery [32], or offered guidance in improving self-perception [47]. Intimate partners, friends, and patients with similar experiences usually respected patients’ autonomy [29, 32, 47, 48]. In addition, patients sometimes sought conversations with others. Rather than requesting specific advice, they aimed to gain external validation for decisions they had already made [41, 46].

*Most of participants said that it was their decision but conferred with others to legitimize their concerns and endorse their treatment-seeking behaviors.* [41] *(p. 254)*

##### Category 12: role of clinicians

Clinicians played a crucial role in surgical decision-making, and providing professional opinions constituted only one aspect. Due to their expertise, clinicians were often regarded as the absolute authorities, and patients placed unconditional trust in them [31, 32, 41, 42]. Clinicians confirmed patients’ concerns and proposed treatment plans [32]. Only a few patients maintained autonomous decision-making when clinicians suggested surgery [31]. In addition, factors such as clinicians’ communication approaches [32, 47], emotional connections [31, 41, 48], and personal experiences [40] also had a considerable impact on patients’ decision-making.

*“They are the expert so I have listened to everything they have said.”* [31] *(p. 260*,* Millie)*

#### Synthesized finding 4: decision timing: why now?

##### Category 13: trigger event

A sudden event could disrupt the surgical decision-making process. A failed romantic relationship [30] or teasing from others [31] could serve as the final straw, prompting patients to make the final decision. Conversely, an unexpected health issue before surgery could force its cancellation [40].

##### Category 14: psychology before surgery

Patients experienced diverse emotions when facing surgery. The decision recurred persistently in their minds [40]. A subset of patients felt guilty, believing the need for surgery was due to their failure to wear braces as instructed [40]. Several held ambivalent perceptions, judging their deformity mild yet still requiring surgical correction [48]. Fearful of the procedure [45], some chose to avoid confronting the issue and neglected essential preoperative preparations [48]. In contrast, other patients viewed surgery as a relief from the desperate situation [32], with excitement outweighing all other concerns [42].

*“Always thinking about it … playing it over in my mind.”* [40] *(p. 119*,* P14)*

### Confidence in review findings

The four synthesized findings align with the descriptive review findings assessed by the GRADE-CERQual approach [38]. Synthesized findings 1, 2, and 3 were assessed as “moderate confidence,” meaning it is likely that the synthesized finding is a reasonable representation of the phenomenon of interest. Synthesized finding 4 was assessed as “low confidence,” meaning it is possible that the synthesized finding is a reasonable representation of the phenomenon of interest. The GRADE-CERQual summary of qualitative findings is provided in Table 4.

**Table 4:** Summary of qualitative findings table

Review finding	CERQual assessment of confidence in the evidence	Explanation of CERQual assessment	Studies contributing to the review finding
Synthesized finding 1: Initial intention: Inner drivers and self-reconstruction	Moderate	Minor concerns regarding methodological limitations (Q6 and Q7 not addressed in most studies) and coherence (alternative treatments). No or very minor concerns regarding adequacy. Moderate concerns regarding relevance (differences in the phenomenon of interest*).	[29–32, 39–48]
Synthesized finding 2: Evaluating pathways: Ideal visions versus practical constraints	Moderate	Minor concerns regarding methodological limitations (Q6 and Q7 not addressed in most studies) and coherence (service experience). No or very minor concerns regarding adequacy. Moderate concerns regarding relevance (differences in the phenomenon of interest*).	[29, 31, 32, 40–42, 45, 48]
Synthesized finding 3: Confirming pathways: Role of social networks	Moderate	Minor concerns regarding methodological limitations (Q6 and Q7 not addressed in most studies). No or very minor concerns regarding coherence and adequacy. Moderate concerns regarding relevance (differences in the phenomenon of interest*).	[29–32, 40–42, 46–48]
Synthesized finding 4: Decision timing: Why now?	Low	Minor concerns regarding methodological limitations (Q6 and Q7 not addressed in most studies) and adequacy (thin data). Moderate concerns regarding coherence (psychology before surgery) and relevance (differences in the phenomenon of interest*).	[30–32, 40, 42, 45, 48]

*Differences in the phenomenon of interest: Three studies [46–48] focused on broader surgical experience rather than specific surgical decision-making

### Robustness checks and cross-contextual comparisons

#### Robustness checks

The 4 synthesized findings and 14 categories remained unchanged after sequentially excluding studies exclusively using social media data [44, 45], a dissertation [32], those with the lowest-quality JBI scores [29, 42], and those focusing on surgical experience rather than surgical decision-making [46–48]. The summary of robustness checks is detailed in Appendix D of the Supplementary Material.

#### Cross-contextual comparisons

By healthcare system, the NHS group [30–32, 39, 40, 43, 46–48] retained 13 of the 14 categories, with category 8 (financial considerations) absent. The non-NHS group [29, 41, 42, 44, 45] retained 12 of the 14 categories, with category 3 (self-schema) and category 13 (trigger event) absent. All 4 synthesized findings remained unchanged in both groups.

By treatment outcome, the surgery-only group [30, 32, 39, 42, 43, 46–48] retained 13 of the 14 categories, with category 8 (financial considerations) absent. The mixed group [29, 31, 40, 41, 44, 45] retained all 14 categories. All 4 synthesized findings remained unchanged in both groups.

The summary of cross-contextual comparisons is detailed in Appendix E of the Supplementary Material.

## Discussion

### Main findings

#### Demand conflict and idealistic expectations

Patients generally just sought to restore normality, whether driven by functional, aesthetic, or psychosocial concerns. These goals appear unremarkable, yet they carry profound significance. The core aspiration of patients is to regain the fundamental right to live without barriers as a full social being. This right has been compromised by their condition.

Patients faced considerable demand conflict. Some explicitly pursued both physical and nonphysical changes. Others claimed to seek only one aspect, yet expectations for the other aspect implicitly emerged in their narratives. Further research showed that patients’ needs before and after treatment were not necessarily the same. Before surgery they tended to prioritize functional improvements, whereas after surgery they expressed expectations regarding appearance changes [25]. Research has clearly demonstrated that orthognathic surgery inherently bridges the gap between function and aesthetics. Correction of skeletal malocclusion necessarily alters facial morphology [49]. This means that even when patients claim to seek exclusively functional improvements, appearance changes cannot be avoided, which may further cause difficulties in adapting to their unfamiliar appearance due to insufficient psychological readiness [50]. This, in turn, may ultimately lead to demands for nonphysical changes. In addition, nonphysical changes are fundamentally dependent upon the physical changes [15]. Patients who claim to desire only nonphysical outcomes implicitly rely on the physical alterations that bring about such psychosocial improvements. It was reported that patients with ambiguous expectations were more likely to be dissatisfied with the surgical outcomes [27]. Therefore, clarification of the needs during consultation is essential. It is important to note that clinicians should identify the underlying types of surgical expectations patients hold, rather than relying solely on their statements.

A minority of patients held overly idealistic expectations that orthognathic surgery alone could bring about a complete transformation in their lives. However, the included primary studies indicated that even after surgery, some patients continued to face social challenges and remained sensitive to evaluations from others [46–48]. Williams et al. also noted that patients seeking orthognathic surgery to improve their social life were less satisfied than those with other motivations [51]. Therefore, patients need to recognize that physical and nonphysical recovery is a gradual process. The reconstruction of self-identity especially takes time. Research has indicated that the permanent effects of orthognathic surgery should be evaluated after 2–5 years [10], and patient satisfaction increases over time [52]. More importantly, the surgery directly alters occlusal function and facial profile [15], whereas how to cope with social reactions requires the active psychological construction [16]. Patients should strive to develop stable inner mechanisms and reduce reliance on external evaluations. It is crucial to trust that they have made the right decision. According to the HBM, the prerequisite for individuals to take action is the perceived threat [33]. Therefore, for patients who have already developed a strong self-identity prior to surgery, the desire for orthognathic surgery may be relatively low.

Despite the limited evidence regarding alternative treatments, clinicians should ensure comprehensive disclosure of all treatment options. This aligns with SDM, which emphasizes respect for patients’ informed preferences [34].

#### From physiological intervention to body-mind integration

The core of patients’ decision-making regarding orthognathic surgery lies in the balance between the perceived benefits and the perceived barriers [33].

Patients expressed anxiety about both insufficient and excessive change after surgery, while a more fundamental concern was whether the surgery could truly resolve their deeper psychosocial problems. The irreversibility of skeletal correction further intensified preoperative anxiety. In addition, previous research has suggested that orthognathic surgery itself may influence patients’ psyche [9]. These findings underscore the need to integrate psychological intervention with surgical treatment, in order to assist patients in achieving concurrent reconstruction of outer appearance and inner world. However, clinical practice appears to remain inadequate in addressing patients’ psychological needs. In the UK, a national survey found that 31.3% of orthodontic units referred no orthognathic patients for psychological assessment at all, primarily due to resource issues [53]. Similarly, a Turkish study reported that, due to a lack of formal training, few orthodontists referred orthognathic patients to psychological support despite acknowledging its importance [54]. There is a gap between awareness and implementation of psychological intervention. Indeed, even successful models have faced prolonged systemic barriers to integrating psychological care into the orthognathic pathway [55].

In addition to the rational assessment of various medical risks, patients considered a range of practical factors. First, this study found that the long treatment duration put life on hold. Previous research in colorectal cancer patients has documented a lack of choice and control in clinical decision-making [56], while our findings suggested that this sense of disempowerment also permeated the broader life context. Second, the high cost of surgery often required the financial commitment of entire family, which transformed what was framed as an individual clinical decision into a collective economic decision. Research on medical debt in Chinese households has demonstrated that such debt could reach multiples of annual household income and constituted a primary driver of poverty [57]. Furthermore, these findings highlighted how patients translated concrete service experience into judgments about healthcare quality. Drawing on Donabedian’s quality framework, structural elements such as environment and equipment serve as visible cues through which patients construct or erode trust in the healthcare institution when they lack technical expertise to evaluate surgical risks directly [58]. Patients’ decision-making is not a simple calculation of benefits versus risks, but a comprehensive consideration shaped by their personal circumstances. As Mapes et al. have argued, achieving truly patient-centered care requires a clearer understanding of how patients’ values are situated within the context of their own lives [59].

#### Diverse influences of social network

This study supports the central role of the clinician-patient relationship in SDM [35], but we found that decision‑making actually occurred within broader social networks. While family members, particularly parents, were often regarded as authoritative figures, their influence was far from uniform. Scholars have noted that parents frequently experienced a tension between managing surgical risks and avoiding social stigma [60]. This conflict may explain such variability of opinions in family members. The role of patients with similar experiences should not be underestimated. On the one hand, such individuals can share treatment information based on personal experience. On the other hand, they can offer patients companionship and encouragement. This finding closely aligns with the three critical attributes of peer support proposed by Dennis: informational support, emotional support, and appraisal support [61]. Friends and other social contacts seldom dictated patients’ decision‑making. Instead, they played a reflective role in patients’ emerging preferences. This is consistent with the social-interdependent choice paradigm, in which peripheral networks primarily help patients clarify and refine their values [62]. We also found that patients did not always seek social support to obtain specific advice on whether to undergo surgery. Their decisions had already been made. This act served primarily to transform their personal choice into social validation. This finding can be explained by social verification theory, which suggests that people seek social consistency with those around them to reduce belongingness threat [63]. Additionally, from the perspective of decision justification theory, this behavior can be understood as an attempt to diffuse decisional responsibility and mitigate anticipated regret [64].

Clinicians were often regarded as absolute authorities. Some patients only became aware of their dentofacial discrepancies after consulting with clinicians. Research has indicated that communication is not merely an information tool but rather a therapeutic intervention [65]. Clinicians should be vigilant about patients’ obsession with their defects and tailor communication approaches accordingly. The present study found that some patients felt they were treated merely as a case without being taken seriously, even when they had received complete professional care from their clinicians. In these situations, clinicians acted only as technical providers. This echoes findings from Auerbach et al., who found that for certain patients, interpersonal interaction with clinicians may be as important as the information conveyed [66]. It follows that how clinicians maintain emotional connections with patients warrants further investigation.

Previous studies suggested that adequate information support was generally associated with higher patient satisfaction [67]. However, this study found that more detailed information was not necessarily better. Personalized information should be provided according to patients’ needs. In addition, visualization tools should be utilized to offer patients more intuitive information, which can help enhance clinician-patient communication, promote realistic expectations, alleviate preoperative anxiety, and strengthen surgical motivation [68].

#### Emotional tipping points in decision-making

In this study, we found that patients’ rational decision-making could be overwhelmed by emotions when confronted with the cues to action [33]. Clinicians should be acutely aware of such situations and determine whether the decision arises from mature deliberation or is made on impulse. Previous research showed that such emotional decisions increased the likelihood of postoperative regret [69]. We observed that the major medical decision was challenging when made, and the complex psychology right before surgery once again highlighted the difficulty of the process. Moon and Kim also reported that preoperative anxiety was common among patients undergoing orthognathic surgery as the procedure approached [70]. The decision-making process reflects a significant psychological adaptation, and the medical team needs to respond accordingly. It should be noted that synthesized finding 4 was assessed as “low confidence” by the GRADE-CERQual approach, and therefore these observations remain exploratory.

#### Differences across contexts

Our cross-contextual comparisons revealed differences across healthcare systems and treatment outcomes. Category 8 (financial considerations) was absent in the NHS group but present in the non-NHS group. Similarly, Felgner et al. reported that out-of-pocket payments were a major barrier to decision-making regarding dental treatments [71], while the NHS exempts patients from these costs [72]. A study conducted in the UK found that orthognathic patients perceived the treatment as “good value for money” and expressed a higher willingness to pay for it [73], suggesting that NHS patients do not frame their decisions in terms of cost. Category 3 (self-schema) and category 13 (trigger event) were absent in the non-NHS group but present in the NHS group. This may be due to the limited number of non-NHS studies. Moreover, all five non-NHS studies employed descriptive qualitative approaches rather than interpretive methodologies such as phenomenology or grounded theory, which may have limited the depth and breadth of these review findings [74]. By treatment outcome, category 8 (financial considerations) appeared only in the mixed group, suggesting that financial considerations may be particularly salient among patients who decline surgery or are undecided. Once patients have committed to surgery, financial considerations are no longer prominent in their narratives. This aligns with the findings of Lerman et al., who reported that 76.4% of patients who declined surgery cited procedure cost as their primary decision factor [75]. Similarly, Farrell and Tucker noted that high hospital charges often caused patients to hesitate about proceeding with orthognathic surgery [76].

### Theoretical integration

The HBM provides the theoretical framework for understanding the decision-making process in this study. However, our findings suggested that patients’ decision-making was phase-based. Moreover, social networks profoundly influenced patients’ decision-making. These aspects are not well captured by the classical theory. While SDM offers a social perspective on the decision-making process, we found that the social networks influencing patients’ decision-making extended beyond the dyadic clinician-patient relationship. Decision-making was deeply embedded in broader social networks that included family members, friends, and patients with similar experiences. These members not only provided support but also altered patients’ decision-making preferences.

**Fig. 2:**
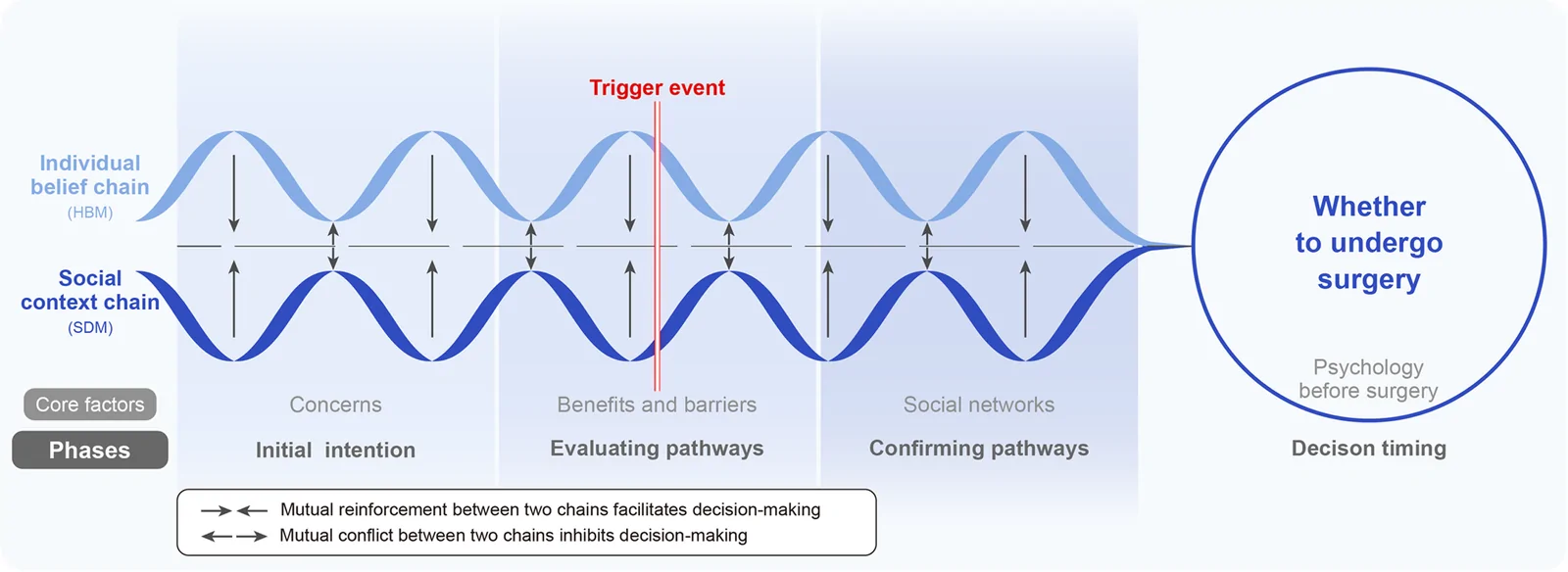
Double-Helix Interactive Decision-Making Model

Based on these findings, we proposed the Double-Helix Interactive Decision-Making Model (DH-IDM) as a hypothesis-generating framework to more comprehensively describe the complex mechanisms underlying patients’ decision-making experiences regarding orthognathic surgery. The structure of this model is presented in Fig. 2.

This model conceptualizes the decision-making process as two interacting helical chains. The first chain, the individual belief chain, is derived from key components of the HBM. It includes perceived threat and behavior evaluation, acting as the psychological driver of decision-making. The second chain, the social context chain, is based on core constructs of SDM. It consists of diverse social networks, serving as the relational field for decision-making. The social context chain continuously influences individual beliefs. In turn, the individual belief chain also continuously shapes social contexts. Mutual reinforcement between the two chains facilitates decision-making, whereas mutual conflict between the two chains inhibits decision-making.

In the initial intention phase, the core factors are patients’ functional, aesthetic, and psychosocial concerns. In some cases, these concerns originate from the social context, and social feedback further strengthens or weakens such concerns. Meanwhile, patients’ coping strategies and personality traits can also amplify or diminish the impact of social evaluation.

In the evaluating pathways phase, the core factors are benefits and barriers. When assessing surgical outcomes, patients partly consider social views on postoperative changes. The perceived barriers directly or indirectly inhibit surgical decision-making. However, patients respond to these barriers in different ways. Some may use cognitive strategies to rationalize the risks, while others may actively work to overcome the obstacles.

In the confirming pathways phase, the core factors are social networks. Specifically, clinicians, family members, friends, and patients with similar experiences exert a concentrated influence on the decision-making. Positive social networks reinforce the patients’ confidence in the decision-making, while negative social networks undermine it. In contrast, patients who have developed the intention to undergo surgery proactively cultivate a supportive environment. Admittedly, a minority of patients decline surgery even when clinicians recommend it.

Finally, as individual beliefs align with social contexts, the two chains stop interacting and become coupled. At this point, patients’ beliefs reach saturation and the process enters the decision timing phase, which leads to the final decision of whether to undergo surgery along with various preoperative psychological states. Alternatively, the interaction between the two chains may be terminated early by trigger events that constitute cues to action.

This model should be viewed as hypothesis-generating rather than confirmatory before rigorous validation. We propose the following validation agenda: (a) cross-cultural qualitative studies to test transferability; (b) cross-sectional studies to test interaction between the two chains; (c) longitudinal studies to test the phase progression from initial intention to decision timing.

### Preliminary blueprint for patient decision aids

Based on the International Patient Decision Aid Standards (IPDAS) [77], this study provided a conceptual framework for the core modules of patient decision aids for orthognathic surgery, as shown in Table 5. It should be noted that this tool remains unvalidated.

**Table 5:** Core modules of patient decision aids for orthognathic surgery

Module	Corresponding review finding	Suggested content in the patient decision aids	Design objective
Preference exploration	Initial intention Category 1–4	Motivation Checklist Select the one or two most relevant motivations for treatment. Expectation Alignment Matrix Physical and nonphysical changes define the horizontal and vertical axes, and patients are anchored as “metamorphosizers,” “pragmatists,” “shedders,” or “evolvers.” Key Information Prompts Provide evidence to guide careful consideration of psychosocial needs. Alternative Options Panel Present a table comparing all treatment options (orthognathic surgery, camouflage orthodontics, and observation) across dimensions such as treatment goals, target population, improvement degree, and major risks.	Why am I here for treatment?
Comprehensive evaluation	Evaluating pathways Category 5–9	Visualization of Outcomes and Risks Show visual simulations of surgical outcomes, and present charts of complication risks and their impacts. Reality Navigator “Which of the following requires the most detailed planning at present?” If treatment duration, consider coordination with study, work, and personal life. If financial considerations, use a cost calculator to estimate out-of-pocket expenses. If service experience, list qualified providers that offer psychological support.	Can I accept and do it?
Social support	Confirming pathways Category 10–12	Clinician-Patient Question List Generate a personalized list of questions based on patient priorities for use during consultation. Support Network Assistance Provide key points for communicating with family members or friends, and link to verified patient communities.	How do I build my support system?
Final decision	Decision timing Category 13–14	Trigger Event Reflection “If this event had not happened, would I still feel the same urgency to consider surgery?” “To what extent does the desire for surgery stem from the emotional response, rather than the condition itself?” Decisional Conflict Self-Assessment Introduce the *Decisional Conflict Scale* [78], provide personalized feedback based on the score, and return to previous modules if necessary. Values Clarification and Expectation Confirmation The *Expectation Confirmation Statement* is automatically generated based on preceding modules, including the single most important expectation and the single most significant concern regarding surgery, to be reviewed with the clinician for final confirmation prior to surgery.	Am I ready?

**Fig. 3:**
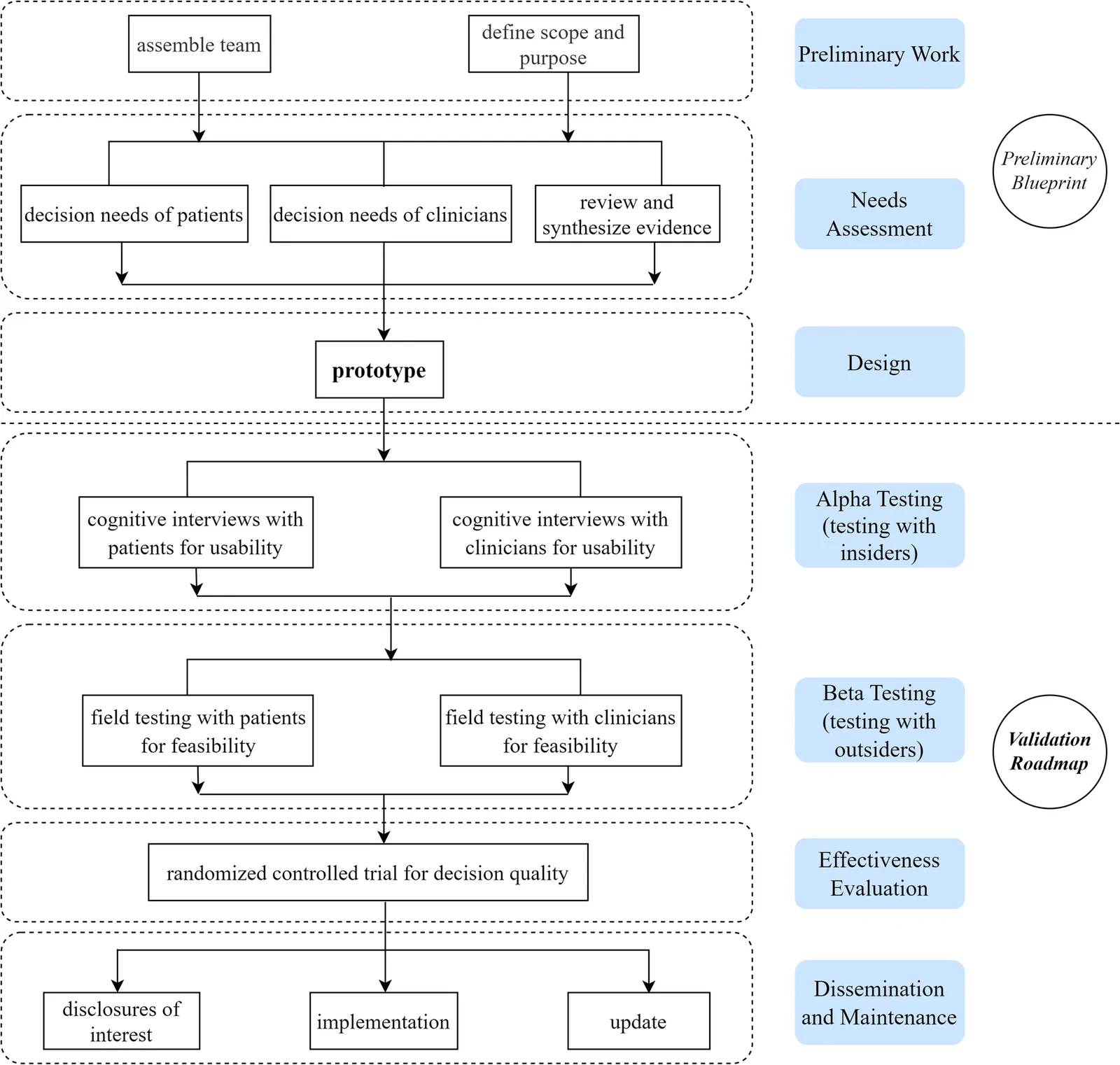
Systematic development process for patient decision aids

Consistent with the IPDAS [77, 79], formal validation is required prior to implementing the patient decision aids for orthognathic surgery in clinical practice. The systematic development process for patient decision aids including the validation roadmap is presented in Fig. 3.

### Recommendations for practice

Faced with the major medical decision of orthognathic surgery, clinical practice should move beyond the technical execution and shift to a patient-centered paradigm for decision-making empowerment. This approach assists patients in achieving informed preferences with adequate information and strong support.

#### Consultation focus: from information provision to needs clarification and expectation management

Needs clarification and expectation management are central to clinician-patient communication. Clinicians can use patient decision aids, such as Motivation Checklist and Expectation Alignment Matrix proposed in this study, to standardize the assessment of patients’ needs for orthognathic surgery. They should also proactively help patients identify treatment expectations that may not be achievable through orthognathic surgery alone. Personalized information and visualization tools can be used to repeatedly align these expectations with surgical outcomes, until mutual understanding is reached and realistic goals are defined.

#### Assessment dimensions: from medical checklist to patients’ entire life

When evaluating the benefits and barriers of orthognathic surgery, it is essential to consider the impact of surgery on the patients’ entire life. Beyond assisting patients in clarifying surgical outcomes and complication risks, clinicians can employ Reality Navigator to help patients anticipate and plan for practical constraints that may hinder surgical decision-making.

#### Support networks: from dyadic clinician-patient interaction to inclusive collaboration

Surgical decision-making is significantly influenced by patients’ social networks. A critical clinical strategy is to facilitate communication between patients and key family members or friends, and cautiously guide patients to verified patient communities. Clinicians should remain alert to patients’ underlying intentions, whether they are exploring options or seeking validation. It is essential for clinicians to refine their communication approaches and cultivate emotional connections with patients throughout the decision-making process.

#### Timely intervention: detection of trigger event and provision of psychological support

When patients exhibit a strong decisional impulse triggered by critical events, clinicians should not respond immediately or forcefully dissuade them. Instead, they can provide tools such as Trigger Event Reflection and Decisional Conflict Self-Assessment to help patients identify their emotional responses and assess decision readiness, thereby facilitating considered decisions. In addition, psychological support should be integrated into the orthognathic pathway from preoperative decision-making to postoperative adaptation, in order to cope with the anxiety associated with major medical decisions and the challenges involved in self-identity reconstruction. This can be achieved through the participation of mental health professionals or by training clinicians in psychology.

### Recommendations for research

This study provides solid evidence for future research by exploring the decision-making experiences of patients with DFD regarding orthognathic surgery. First, further research should quantify the weight of influence exerted by individual beliefs and social contexts on patients’ decision-making. Long-term follow-up is also warranted to track the predictive value of major factors influencing decision-making for patients’ quality of life. Second, we recommend developing and validating a localized decision-making assessment scale for orthognathic surgery, so as to facilitate large-scale population surveys. Third, the exploration of patient decision aids based on artificial intelligence has dual significance. Such technology can not only standardize the decision-making support pathway but also enable clinical staff to shift from repetitive tasks to emotional support. Finally, it is necessary to establish a clinical decision support system for orthognathic surgery, and implementation research should be carried out to promote the translation of evidence into practice.

### Limitations

Several limitations of this meta-synthesis should be acknowledged. Two studies exclusively using social media data lacked formal ethical approval and individual informed consent, a recognized challenge in internet-based research. They also differed from interview-based studies in content richness, data depth, and researcher-participant interaction. We did not systematically search for grey literature, which may have resulted in the omission of relevant studies. Studies with the lowest‑quality JBI scores were included, while they exhibited methodological shortcomings. Studies focusing on surgical experience rather than surgical decision-making differed from our phenomenon of interest, although decision-relevant data could still be extracted. Despite these limitations, robustness checks confirmed that their inclusion did not introduce critical bias into this study. Notably, two included studies were conducted by the same authors using overlapping samples, which may have overrepresented certain perspectives. Moreover, we only included studies in Chinese or English. Relevant studies in other languages may have been missed. This language bias may restrict the cross-cultural applicability of our findings. Most studies were conducted within the NHS in the UK, which may limit the transferability of the review findings to lower-resource or private-practice settings. More than half of the studies included only patients who had decided to undergo or had already undergone surgery, while other studies included mixed samples without reporting data separately. Therefore, the review findings should be interpreted with caution when applied to patients who decline surgery or are undecided.

## Conclusions

Patients with DFD experience a complex decision-making process embedded in individual beliefs and social contexts. The process begins with the drive of inner needs. It then involves a rational assessment of benefits and barriers. External networks exert continuous influence on patients’ decision-making. Ultimately, action may be taken in response to trigger events. This study proposes a novel DH-IDM and provides a preliminary conceptual framework for patient decision aids. It also calls for a shift in clinical practice from technical execution to decision-making empowerment. Due to limitations in the primary studies, the review findings require further validation in broader samples.

## Supplementary Information

Supplementary Material 1.

## Data Availability

All data generated or analyzed during this study are included in this published article and its supplementary material.
