# Supplementary material for: Elective orthognathic surgery: a meta-synthesis of patients’ decision-making experiences
Source: BMC Oral Health. 2026 Jul 29;26:2198. doi: 10.1186/s12903-026-09066-z (PMC13741441; doi:10.1186/s12903-026-09066-z)
Supplement: Supplementary file 1 [file 12903_2026_9066_MOESM1_ESM.docx]

**Appendix A: Search strategy**

**PubMed MEDLINE-546**

| Search number | Query | Results |
| --- | --- | --- |
| 1 | "Orthognathic Surgery"[Mesh] | 1,327 |
| 2 | (((orthognathic[Title/Abstract]) OR (orthognathic operation[Title/Abstract])) OR (orthognathic treatment[Title/Abstract])) OR (jaw surgery[Title/Abstract]) | 7,891 |
| 3 | #1 OR #2 | 8,181 |
| 4 | "Decision Making"[Mesh] | 240,154 |
| 5 | ((((((((((((((((((decision[Title/Abstract]) OR (decide[Title/Abstract])) OR (choice[Title/Abstract])) OR (choose[Title/Abstract])) OR (option[Title/Abstract])) OR (opt[Title/Abstract])) OR (resolution[Title/Abstract])) OR (resolve[Title/Abstract])) OR (select*[Title/Abstract])) OR (elect*[Title/Abstract])) OR (judg*[Title/Abstract])) OR (accept*[Title/Abstract])) OR (refus*[Title/Abstract])) OR (determin*[Title/Abstract])) OR (intent*[Title/Abstract])) OR (motiv*[Title/Abstract])) OR (expect*[Title/Abstract])) OR (concern*[Title/Abstract])) OR (need[Title/Abstract]) | 11,753,298 |
| 6 | #4 OR #5 | 11,852,277 |
| 7 | "Qualitative Research"[Mesh] | 90,977 |
| 8 | ((((((((((((qualitative[Title/Abstract]) OR (grounded theory[Title/Abstract])) OR (phenomenolog*[Title/Abstract])) OR (ethnograph*[Title/Abstract])) OR (case study[Title/Abstract])) OR (interview*[Title/Abstract])) OR (narrative*[Title/Abstract])) OR (belief*[Title/Abstract])) OR (attitude*[Title/Abstract])) OR (feeling*[Title/Abstract])) OR (emotion*[Title/Abstract])) OR (perception*[Title/Abstract])) OR (experience*[Title/Abstract]) | 2,783,803 |
| 9 | #7 OR #8 | 2,788,260 |
| 10 | #3 AND #6 AND #9 | 546 |

**Ovid MEDLINE-536**

| Search number | Query | Results |
| --- | --- | --- |
| 1 | (orthognathic surgery or orthognathic or orthognathic operation or orthognathic treatment or jaw surgery).ti,ab,kw. | 7853 |
| 2 | (decision making or decision or decide or choice or choose or option or opt or resolution or resolve or select* or elect* or judg* or accept* or refus* or determin* or intent* or motiv* or expect* or concern* or need).ti,ab,kw. | 11722597 |
| 3 | (qualitative research or qualitative or grounded theory or phenomenolog* or ethnograph* or case study or interview* or narrative* or belief* or attitude* or feeling* or emotion* or perception* or experience*).ti,ab,kw. | 2775145 |
| 4 | 1 and 2 and 3 | 536 |

**Embase-1053**

| Search number | Query | Results |
| --- | --- | --- |
| 1 | 'orthognathic surgery'/exp | 13910 |
| 2 | 'orthognathic':ab,ti OR 'orthognathic operation':ab,ti OR 'orthognathic treatment':ab,ti OR 'jaw surgery':ab,ti | 8459 |
| 3 | #1 OR #2 | 16898 |
| 4 | 'decision making'/exp | 496756 |
| 5 | 'decision':ab,ti OR 'decide':ab,ti OR 'choice':ab,ti OR 'choose':ab,ti OR 'option':ab,ti OR 'opt':ab,ti OR 'resolution':ab,ti OR 'resolve':ab,ti OR 'select*':ab,ti OR 'elect*':ab,ti OR 'judg*':ab,ti OR 'accept*':ab,ti OR 'refus*':ab,ti OR 'determin*':ab,ti OR 'intent*':ab,ti OR 'motiv*':ab,ti OR 'expect*':ab,ti OR 'concern*':ab,ti OR 'need':ab,ti | 14845618 |
| 6 | #4 OR #5 | 15007243 |
| 7 | 'qualitative research'/exp | 132029 |
| 8 | 'qualitative':ab,ti OR 'grounded theory':ab,ti OR 'phenomenolog*':ab,ti OR 'ethnograph*':ab,ti OR 'case study':ab,ti OR 'interview*':ab,ti OR 'narrative*':ab,ti OR 'belief*':ab,ti OR 'attitude*':ab,ti OR 'feeling*':ab,ti OR 'emotion*':ab,ti OR 'perception*':ab,ti OR 'experience*':ab,ti | 3686753 |
| 9 | #7 OR #8 | 3695204 |
| 10 | #3 AND #6 AND #9 | 1053 |

**CINAHL-116**

| Search number | Query | Results |
| --- | --- | --- |
| 1 | MH "Orthognathic Surgery" OR TI ("orthognathic" or "orthognathic operation" or "orthognathic treatment" or "jaw surgery") OR AB ("orthognathic" or "orthognathic operation" or "orthognathic treatment" or "jaw surgery") | 1725 |
| 2 | MH "Decision Making" OR TI ("decision" or "decide" or "choice" or "choose" or "option" or "opt" or "resolution" or "resolve" or "select*" or "elect*" or "judg*" or "accept*" or "refus*" or "determin*" or "intent*" or "motiv*" or "expect*" or "concern*" or "need") OR AB ("decision" or "decide" or "choice" or "choose" or "option" or "opt" or "resolution" or "resolve" or "select*" or "elect*" or "judg*" or "accept*" or "refus*" or "determin*" or "intent*" or "motiv*" or "expect*" or "concern*" or "need") | 2050392 |
| 3 | MH "Qualitative Studies" OR TI ("qualitative" or "grounded theory" or "phenomenolog*" or "ethnograph*" or "case study" or "interview*" or "narrative*" or "belief*" or "attitude*" or "feeling*" or "emotion*" or "perception*" or "experience*") OR AB ("qualitative" or "grounded theory" or "phenomenolog*" or "ethnograph*" or "case study" or "interview*" or "narrative*" or "belief*" or "attitude*" or "feeling*" or "emotion*" or "perception*" or "experience*") | 1107716 |
| 4 | S1 AND S2 AND S3 | 116 |

**Scopus-826**

( TITLE-ABS-KEY ( "orthognathic surgery" OR "orthognathic" OR "orthognathic operation" OR "orthognathic treatment" OR "jaw surgery" ) AND TITLE-ABS-KEY ( "decision making" OR "decision" OR "decide" OR "choice" OR "choose" OR "option" OR "opt" OR "resolution" OR "resolve" OR "select*" OR "elect*" OR "judg*" OR "accept*" OR "refus*" OR "determin*" OR "intent*" OR "motiv*" OR "expect*" OR "concern*" OR "need" ) AND TITLE-ABS-KEY ( "qualitative research" OR "qualitative" OR "grounded theory" OR "phenomenolog*" OR "ethnograph*" OR "case study" OR "interview*" OR "narrative*" OR "belief*" OR "attitude*" OR "feeling*" OR "emotion*" OR "perception*" OR "experience*" ) )

**ProQuest-151**

| Search number | Query | Results |
| --- | --- | --- |
| 1 | TI,AB("orthognathic surgery" or "orthognathic" or "orthognathic operation" or "orthognathic treatment" or "jaw surgery") | 1597 |
| 2 | TI,AB("decision making" or "decision" or "decide" or "choice" or "choose" or "option" or "opt" or "resolution" or "resolve" or "select*" or "elect*" or "judg*" or "accept*" or "refus*" or "determin*" or "intent*" or "motiv*" or "expect*" or "concern*" or "need") | 34734606 |
| 3 | TI,AB("qualitative research" or "qualitative" or "grounded theory" or "phenomenolog*" or "ethnograph*" or "case study" or "interview*" or "narrative*" or "belief*" or "attitude*" or "feeling*" or "emotion*" or "perception*" or "experience*") | 6729633 |
| 4 | [S1] AND [S2] AND [S3] | 151 |

**Web of Science-4551**

| Search number | Query | Results |
| --- | --- | --- |
| 1 | TS=(orthognathic surgery OR orthognathic OR orthognathic operation OR orthognathic treatment OR jaw surgery) and Preprint Citation Index (Exclude – Database) | 42539 |
| 2 | TS=(decision making OR decision OR decide OR choice OR choose OR option OR opt OR resolution OR resolve OR select* OR elect* OR judg* OR accept* OR refus* OR determin* OR intent* OR motiv* OR expect* OR concern* OR need) and Preprint Citation Index (Exclude – Database) | 68948045 |
| 3 | TS=(qualitative research OR qualitative OR grounded theory OR phenomenolog* OR ethnograph* OR case study OR interview* OR narrative* OR belief* OR attitude* OR feeling* OR emotion* OR perception* OR experience*) and Preprint Citation Index (Exclude – Database) | 16271026 |
| 4 | #1 AND #2 AND #3 and Preprint Citation Index (Exclude – Database) | 4551 |

**Cochrane Library-236**

| Search number | Query | Results |
| --- | --- | --- |
| 1 | MeSH descriptor: [Orthognathic Surgery] explode all trees | 89 |
| 2 | (orthognathic):ti,ab,kw or (orthognathic operation):ti,ab,kw or (orthognathic treatment):ti,ab,kw or (jaw surgery):ti,ab,kw | 2535 |
| 3 | #1 or #2 | 2535 |
| 4 | MeSH descriptor: [Decision Making] explode all trees | 6532 |
| 5 | (decision):ti,ab,kw or (decide):ti,ab,kw or (choice):ti,ab,kw or (choose):ti,ab,kw or (option):ti,ab,kw or (opt):ti,ab,kw or (resolution):ti,ab,kw or (resolve):ti,ab,kw or (select*):ti,ab,kw or (elect*):ti,ab,kw or (judg*):ti,ab,kw or (accept*):ti,ab,kw or (refus*):ti,ab,kw or (determin*):ti,ab,kw or (intent*):ti,ab,kw or (motiv*):ti,ab,kw or (expect*):ti,ab,kw or (concern*):ti,ab,kw or (need):ti,ab,kw | 882361 |
| 6 | #4 or #5 | 882839 |
| 7 | MeSH descriptor: [Qualitative Research] explode all trees | 2345 |
| 8 | (qualitative):ti,ab,kw or (grounded theory):ti,ab,kw or (phenomenolog*):ti,ab,kw or (ethnograph*):ti,ab,kw or (case study):ti,ab,kw or (interview*):ti,ab,kw or (narrative*):ti,ab,kw or (belief*):ti,ab,kw or (attitude*):ti,ab,kw or (feeling*):ti,ab,kw or (emotion*):ti,ab,kw or (perception*):ti,ab,kw or (experience*):ti,ab,kw | 339521 |
| 9 | #7 or #8 | 339521 |
| 10 | #3 and #6 and #9 | 236 |

**SinoMed-472**

| Search number | Query | Results |
| --- | --- | --- |
| 1 | "正颌外科手术"[不加权:扩展] | 1205 |
| 2 | "正颌"[常用字段:智能] OR "正颌外科"[常用字段:智能] OR "正颌手术"[常用字段:智能] OR "正颌治疗"[常用字段:智能] | 2522 |
| 3 | (#2) OR (#1) | 2715 |
| 4 | "决策"[不加权:扩展] | 13370 |
| 5 | "决定"[常用字段:智能] OR "决心"[常用字段:智能] OR "抉择"[常用字段:智能] OR "选择"[常用字段:智能] OR "判断"[常用字段:智能] OR "接受"[常用字段:智能] OR "拒绝"[常用字段:智能] OR "确定"[常用字段:智能] OR "目的"[常用字段:智能] OR "意图"[常用字段:智能] OR "动机"[常用字段:智能] OR "期望"[常用字段:智能] OR "需求"[常用字段:智能] | 7032246 |
| 6 | (#5) OR (#4) | 7036602 |
| 7 | "定性研究"[不加权:扩展] | 6502 |
| 8 | "定性"[常用字段:智能] OR "质性"[常用字段:智能] OR "扎根理论"[常用字段:智能] OR "现象学"[常用字段:智能] OR "人种学"[常用字段:智能] OR "民族志"[常用字段:智能] OR "个案研究"[常用字段:智能] OR "访谈"[常用字段:智能] OR "叙事"[常用字段:智能] OR "信念"[常用字段:智能] OR "态度"[常用字段:智能] OR "情感"[常用字段:智能] OR "情绪"[常用字段:智能] OR "感受"[常用字段:智能] OR "体验"[常用字段:智能] OR "经历"[常用字段:智能] OR "经验"[常用字段:智能] | 2983691 |
| 9 | (#8) OR (#7) | 2983709 |
| 10 | (#9) AND (#6) AND (#3) | 472 |

**CNKI-11**

（主题：正颌外科手术 + 正颌 + 正颌外科 + 正颌手术 + 正颌治疗）AND（主题：决策 + 决定 + 决心 + 抉择 + 选择 + 判断 + 接受 + 拒绝 + 确定 + 目的 + 意图 + 动机 + 期望 + 需求）AND（主题：定性研究 + 定性 + 质性 + 扎根理论 + 现象学 + 人种学 + 民族志 + 个案研究 + 访谈 + 叙事 + 信念 + 态度 + 情感 + 情绪 + 感受 + 体验 + 经历 + 经验）

资源范围：总库; 中英文扩展; 时间范围：更新时间：不限;

2024-08-02 22:42:28

**Wanfang-319**

主题:("正颌外科手术" OR "正颌" OR "正颌外科" OR "正颌手术" OR "正颌治疗") AND 主题:("决策" OR "决定" OR "决心" OR "抉择" OR "选择" OR "判断" OR "接受" OR "拒绝" OR "确定" OR "目的" OR "意图" OR "动机" OR "期望" OR "需求") AND 主题:("定性研究" OR "定性" OR "质性" OR "扎根理论" OR "现象学" OR "人种学" OR "民族志" OR "个案研究" OR "访谈" OR "叙事" OR "信念" OR "态度" OR "情感" OR "情绪" OR "感受" OR "体验" OR "经历" OR "经验")

**VIP-2**

((((((题名或关键词=正颌外科手术 OR 题名或关键词=正颌) OR 题名或关键词=正颌外科) OR 题名或关键词=正颌手术) OR 题名或关键词=正颌治疗) AND (((((((((((((题名或关键词=决策 OR 题名或关键词=决定) OR 题名或关键词=决心) OR 题名或关键词=抉择) OR 题名或关键词=选择) OR 题名或关键词=判断) OR 题名或关键词=接受) OR 题名或关键词=拒绝) OR 题名或关键词=确定) OR 题名或关键词=目的) OR 题名或关键词=意图) OR 题名或关键词=动机) OR 题名或关键词=期望) OR 题名或关键词=需求)) AND (((((((((((((((((题名或关键词=定性研究 OR 题名或关键词=定性) OR 题名或关键词=质性) OR 题名或关键词=扎根理论) OR 题名或关键词=现象学) OR 题名或关键词=人种学) OR 题名或关键词=民族志) OR 题名或关键词=个案研究) OR 题名或关键词=访谈) OR 题名或关键词=叙事) OR 题名或关键词=信念) OR 题名或关键词=态度) OR 题名或关键词=情感) OR 题名或关键词=情绪) OR 题名或关键词=感受) OR 题名或关键词=体验) OR 题名或关键词=经历) OR 题名或关键词=经验)

**Appendix B: Study findings and illustrations**

**HU X, Xiao Y, Wang B, Ruan H, Yuan W. Qualitative study on the decision-making of orthognathic surgery in adult patients with malocclusion. Shanghai Kou Qiang Yi Xue. 2019;28(5):513-7.**

| Findings | Illustration from Publication (page number) | Evidence |
| --- | --- | --- |
| Aesthetic needs | A1: “I know I have a skeletal problem. Only orthognathic surgery can fix it. I am really scared inside, but I also want to become beautiful. I am caught in endless mental struggle.” A3: “My side profile looks like that of a Peking Man. It is like I have not evolved properly. The sooner it gets better, the better.” A4: “When I was a kid, I did not care. But now I have higher standards for my looks. I always feel that I do not look good.” A5: “I have a small chin and small eyes. My whole face looks ugly, so I have just endured it. In the last few years, I feel so ugly that I cannot stand it anymore. I want to improve myself, but after all, this surgery is cosmetic surgery.” A6: “I have too many troubles. I do not dare to smile. My side face is ugly. What I care about most is improving my side profile.” A7: “I just feel that my problem is really serious. It is not just an underbite. My face is also tilted a lot to the left. I have never dared to take a front-facing photo. I hope that after treatment there will be some improvement, and that I will look better than I do now.” A8: “I wore braces when I was a child, but now no one can tell I had them. My problem will not change much with orthodontics alone. To become normal, I need orthognathic surgery.” A9: “I have no chin, which makes my mouth look protruding. When I take photos, I will subconsciously cover my chin.” A10: “When I look at my mouth, I think it is so ugly, especially when I laugh hard. That is why I never show my teeth in photos.” (p. 515) | Unequivocal |
| Psychological needs | A1: “If I do not have the surgery, I feel uncomfortable mentally. Many people ask me out of curiosity why I am like this. I do not know how to answer, and that has made me more introverted in social interactions at university.” A2: “Every time I look in the mirror, I feel very upset. I feel especially depressed. If I do not even like myself, how can others like me?” A4: “I make noise when I sleep. My roommates dislike me. I have been extremely insecure since I was a child. I really want to become handsome.” A5: “I feel abnormal. I have very little confidence. I just want to be like a normal person.” A6: “I have always cared a lot about my moon face problem. I envy normal people. No matter whether they are good-looking or not, at least their overall facial bone structure is fine. Why am I different from others?” A8: “I do not like it when my appearance problem becomes the focus of my colleagues’ discussions. It makes me feel insecure and lonely.” A9: “I am always paying attention to what others mean by their gaze. I do not know if it is discrimination because of my looks. It is too hard to accept my own imperfections. I hope that after surgery I will not have to worry about the meaning behind other people’s gazes, and that I can laugh happily.” (p. 515) | Unequivocal |
| Functional improvement | A3: “Bad occlusion affects me too much. I cannot chew food well, which leads to malnutrition. My stomach is not good either.” A4: “Right now, I am really both ugly and inconvenient. I also make noise when I sleep at night.” A7: “A doctor I saw before said my occlusion is incorrect. The problem will get worse as I get older. It may cause inflammation of the jaw joint and other issues. I want to solve it as early as possible.” (p. 515) | Unequivocal |
| Financial situation | A2: “The doctor talked to me about the cost. My family can fully cover it, and they are also supportive financially, so I decided to go ahead.” A3: “The cost is actually fine. It does not affect me much.” A5: “The treatment cost is very high. Medical insurance cannot cover part of it. I can only wait until I save enough money before doing it.” A6: “Maybe I am just stuck like this for life. What can I do? I am broke and ugly. I feel like a loser every day.” A8: “Cost is not a big problem. I can still afford it.” A9: “Surgery is expensive, but I have no choice. The fee is very high. I work hard to earn money. When I have enough money, I will do it.” A10: “The treatment cost is too high. For my family, this is also a big expense. Right now, the burden is too heavy.” (p. 515) | Unequivocal |
| Surgical safety | A1: “I know this is major surgery with high risk, so I am also very scared. After all, it involves operating on bones, so I thought about it for a long time.” A3: “I know there might be nerve damage or anesthesia accidents. But any surgery has risks and complications. If you do not do it now, you do not know what will happen later.” A4: “Actually, I am not afraid of surgery at all. As long as I can become normal, any pain is worth it. Even if there are risks, I have to bear them. Everything comes at a price.” A5: “If the surgery is performed at a major hospital renowned for orthognathic surgery, the surgical risks should not be too high.” A9: “This is not a small surgery. I consulted the doctor. He said the surgery is definitely mature. But after all, it is general anesthesia. All the risks that come with general anesthesia will still be there, and they cannot be prevented. I am still a bit worried psychologically.” (p. 515) | Unequivocal |
| Family support | A1: “My parents absolutely disagree, and I do not have money. Orthognathic surgery is expensive. I do not know when I will be able to save enough.” A2: “My family thinks that as long as the surgery is mature, it is okay to do it.” A3: “The doctor said I need to have teeth extracted. My mother did not agree. To persuade her to approve the surgery will take a lot of work.” A4: “After discussing with my family, I received their full support.” A5: “My parents think I already look pretty good. They do not think I need improvement, and they definitely do not want me to have surgery.” A7: “My mother did a lot of research. She always hopes I can become a bit more normal, so they are willing to share the risk with me.” A8: “My wife does not object strongly. She also knows I have always wanted to improve, so overall she is supportive.” A10: “My family does not approve. They said my husband must agree after I get married.” (pp. 515-516) | Unequivocal |
| Information services | A1: “I learned about the surgery mostly from online forums. Doctors did not say much. I always want to ask everything clearly before I decide.” A3: “I did not know before that I need orthodontics before and after the surgery, and that it would take so long. Maybe I cannot do it.” A4: “I read many bloggers’ posts. They say orthognathic surgery is really a terrifying operation, and the recovery is very painful. After the face-to-face consultation, I hope to get more information I want from the doctor, so I can make a decision.” A6: “In other aspects, I actually have no problem. I had already decided to do it, but recently the doctor told me that I might need a blood transfusion during the operation. I cannot accept that, so I still will not have this surgery.” A9: “I just do not trust information from others. I need to hear it directly from my doctor or see the official notice. Only then can I decide.” (p. 516) | Unequivocal |
| Surgical outcomes | A3: “I was planning to do it before, but after listening to the doctor, the degree of improvement is not very big. I want to see if there are other options that can do better before I decide.” A4: “Of course I hope to become beautiful after surgery, but I also worry that there may be some bad facial effects afterwards, especially when I get older.” A5: “I am worried about leftover problems after surgery. My problem is quite severe. I do not know if relapse will happen.” A9: “Technology is so advanced now. Can we not use a computer to design and show me what I will look like after surgery? That way I can know what the result will be, and it will be easier for me to make up my mind.” A10: “I just do not know if the surgery can turn out the way I imagine.” (p. 516) | Unequivocal |

**Ryan FS, Barnard M, Cunningham SJ. Impact of dentofacial deformity and motivation for treatment: a qualitative study. Am J Orthod Dentofac Orthop. 2012;141(6):734-42.**

| Findings | Illustration from Publication (page number) | Evidence |
| --- | --- | --- |
| Initial awareness of problem | “It bothers me because once you know about it you can’t help noticing it (patient 13).” “I just thought there was something about my face … and I thought it was my nose … and then they told me that my lower jaw was growing too far forward. I then … realized hang on, no my nose is fine, it’s my lower, it’s this part of my mouth I don’t like, and it just became a kind of fixation (patient 8).” (p. 736) | Unequivocal |
| Nature of the problem | “I had kids making fun of that (my teeth), I also had kids making fun of my cast arm, I also had kids making fun of my haircuts that my mum used to make me have and to be honest, it never really bothered me because, I never let that sort of thing bother me. I’m more worried about the impact it’s going to have on my eating habits than anything else (patient 13).” “Sometimes I get so emotional and just sit down and look at in the mirror and I’m like what’s going on, what happened to me (patient 3).” (p. 737) | Unequivocal |
| Impact on interpersonal relationships | Some patients avoided socializing (Patient 11) (Patient 7). There were senses of paranoia (patient 18) and insecurity (patients 8 and 14), and feeling defensive (patient 1) because of concerns that others were making judgments based on their appearance. Some were bullied as children because of their appearance (patients 11 and 14). Problems forming intimate relationships were also described (patients 9 and 14). Some participants had been teased by siblings in childhood (patients 4, 6, and 18), and this had led to problems with interfamily relationships when they took their frustrations out on those close to them or put up barriers such as avoiding family gatherings (patients 6, 7, 14, and 18). (p. 737) | Unequivocal |
| Impact on employment | “I’m not achieving what I should be, and that’s one of the reasons, I don’t like public standing up and doing public speaking, I find it difficult to voice my opinions in groups (patient 14).” “I avoid face-to-face communication with people. I always had a very good salaried paid job, but I moved out from it because I was very uncomfortable ... there, so now I do work in the warehouse, so I’m always at the back (patient 7).” “When I’m in an interview say and there’s two girls and me ... they’re not gonna choose me because of my look. I was gonna be a like presenter and they like said you struggle with your talking and your look doesn’t look on the um on the camera basically very good so they got someone else ... that was so horrible (patient 3).” (pp. 737-738) | Unequivocal |
| Impact on confidence | Negative self-image ranged from mild dislike of the appearance of the teeth and face to a sense of self-loathing, with people using expressions such as “deformed,” “freak,” and “damaged inside.” There was a sense of having something wrong with them. In some subjects, the impacts on self-confidence were so strong that they caused a low mood and even depression (patients 6, 7, and 14). (p. 738) | Unequivocal |
| How the problem makes them feel | There was a feeling of victimization, “why me,” of being singled out and punished unfairly, and not knowing why. Some expressed feeling let down and looked down on as a result (patient 6). There was a general feeling that life was more difficult and might have turned out differently if they had not been affected in this way, that they had an additional hurdle to jump or a “millstone” around their neck (patient 14). (p. 738) | Unequivocal |
| Coping strategies | Avoidance coping behavior included not going out (patient 2), not socializing, not eating in front of people (patient 14), not having photographs taken (patient 14), not smiling (patient 4), not thinking about the problem, not allowing friends or family to discuss it, and not looking in the mirror (patient 6). An altered coping strategy involved a huge range of altered behaviors that included covering the mouth to hide the teeth, not biting together to hide the bite (patients 4 and 10), not posing for photos straight on (patient 9), keeping the mouth open (patient 17), retracting the jaw (patient 14), mirror checking with posing on their best side to reassure themselves, putting the tongue under the top lip so it looked fuller (patient 14), moving or blinking during photographs so the photo would be ruined and not be put in the album, posturing the jaw forward (patient 11), and positioning strategies so that people were unable to view them in profile (patient 3). (p. 738) | Unequivocal |
| Trigger for treatment now | “From the moment I heard about it, there was some sort of a light bulb went on up there and I just was happy. It’s, like, you know, you having some sort of illness and finding there’s a cure for it, so that’s the kind of thing. I never knew there was a cure for it, so I was really, really happy about it (patient 13).” “A failed relationship (was a trigger for seeking treatment), and I thought the reasons for it were to do with my self-esteem and my behavior related to this, and I thought I ought to do something about it (patient 14).” “My facial expression changed in a way because I’m not sure if my lower jaw recessed or what it was but I did notice a massive difference ... between the lower jaw and the top jaw was a wake-up call (patient 13).” (p. 739) | Unequivocal |
| Reason for wanting treatment | “I don’t like where I’m going at the moment, and that’s why I’m doing something about it, because I want my life to change, because I want to be out there, I want to be with the outside crowd, I want to go out, I want to talk to people, I want to be friendly with my colleagues and everyone. That’s everything that I want to and, at the moment, the only thing that’s stopping me from doing so is just my teeth (patient 7).” (p. 739) | Unequivocal |
| Source of motivation | “Once it starts interfering with my eating habits which I take religiously, I’m very serious about my, I love to eat, I eat a lot, I’m known for that and you know anything that would have an impact on that will annoy me (patient 13).” (p. 739) | Unequivocal |

**Ryan FS, Barnard M, Cunningham SJ. What are orthognathic patients’ expectations of treatment outcome - a qualitative study. J Oral Maxillofac Surg. 2012;70(11):2648-55.**

| Findings | Illustration from Publication (page number) | Evidence |
| --- | --- | --- |
| Functional or appearance expectations | “I expect my face like won’t change, hopefully. I expect my teeth to bite properly and ... that I should be able to bite properly for the rest of my life.” “I just wanna to have a normal jaw in size and place. I don’t expect so much difference, I don’t but I just want to have a proper jaw that my lips are properly closing.” (pp. 2650-2651) | Unequivocal |
| Emotional effects | “That will actually change my life big time ... I will be the happiest.” (p. 2651) | Unequivocal |
| Social effects | “Because the only thing that now stops me from putting myself out there is just about my teeth. So as long as they’re fixed, I’ll try to be more sociable and more engaging with people.” (p. 2651) | Unequivocal |
| Psychological effects | “Just being able to relax and forget about your appearance, it’s me being happy with my appearance and saying okay, that’s all okay now, I don’t have to think about that, and I’ll just get on with everything and be like everybody else, because I’ve seen what everybody else is like.” “I won’t be ... putting myself down as much as I am and stopping myself to do stuff because of my teeth. I won’t be ... such ... of a critic with myself.” (p. 2651) | Unequivocal |
| Lifestyle effects | “I think I will be, you know, happy (laughs). I would look myself maybe in the mirror, you know go out and you know just enjoy life just like everybody else because right now it just ... this is just like more like kind of, yeah it’s actually affecting my life a lot of ways.” (p. 2651) | Unequivocal |
| Metamorphosizers | Case study: P6 expects treatment to change her life “big time” and she will look “way better.” She will then be able to “enjoy life, like everybody else.” People will not look down on her any more. She hopes that she will be unrecognizable after treatment as a friend of her mother’s had it done and people did not recognize her. Her mother thinks this will be the case and so does she. She thinks her lips will be “perfect” afterwards and her appearance will be 10 out of 10. (pp. 2651-2652) | Unequivocal |
| Pragmatists | Case study: P15 just wants to be able to eat properly. He always noticed he could not chew well and had to chew and swallow big chunks of food. He has to have his steak well done instead of medium rare, as he prefers, so he can chew and digest it. He gets full quite quickly as he has to eat slowly but then he is hungry again an hour later. He also just bites on the back teeth and that is annoying and may wear the teeth down. He just wants his teeth to come together better and in the correct relationship. He does not expect the treatment to have any change on his life in general as “it’s just teeth.” (p. 2652) | Unequivocal |
| Shedders | Case study: P9 just wants to look “a bit more normal.” She thinks if she has the treatment she will have more confidence and will go out more and do more things. She will be more talkative and sociable and meet new people. She feels this will really help with her career in the music industry as she will have the confidence to put herself “out there” and will be more successful. She also thinks her jaw affects the way she sings and treatment will improve that and allow her to sing higher notes without strain. She notices the physical defect and has wondered if it makes her “less pretty” but has come to terms with it and accepts it. However it is nice to know now that something can be done about it. She expects her jaw to be a bit more symmetrical after treatment. (p. 2652) | Unequivocal |
| Evolvers | Case study: P5 admits he probably wouldn’t have pushed for the treatment if his dentist had not motivated him. He noticed the problem in his teens when he could not bite sellotape and observed that his front teeth did not come together but it was his dentist who suggested having treatment to correct it. His mother is very keen for him to have the treatment as she has had a lot of problems with her teeth. Now that the problem has been pointed out to him, he admits it can be annoying when he cannot bite properly and now he is very motivated to have the treatment. He just wants to have “straight teeth and bite properly.” “I expect my face like won’t change hopefully but if it makes me even more good looking I ain’t gonna complain about that, I just expect my teeth to bite properly.” He gets a little frustrated when eating takes so long and it’s embarrassing sometimes in front of others as he is messy when he is eating. He sometimes feels stupid because he cannot eat certain things. He thinks he will be a bit more confident and smile more and show his teeth after treatment. He thinks he may make a better first impression on people, for example at interviews, if his teeth and smile are nice. (p. 2652) | Unequivocal |

**Stirling J, Latchford G, Morris DO, Kindelan J, Spencer RJ, Bekker HL. Elective orthognathic treatment decision making: a survey of patient reasons and experiences. J Orthod. 2007;34(2):113-27.**

| Findings | Illustration from Publication (page number) | Evidence |
| --- | --- | --- |
| Motives for seeking orthognathic treatment | “I wanted to be able to laugh without covering my mouth and I thought others were talking about my teeth. (patient 58)” “I always blamed everything on the fact I looked funny. I suppose it’s only part of the picture. (patient 84)” “Facial appearance has been there always really…in my teens it was a big thing, I hated it. (patient 62)” “I was teased quite a lot when I was younger over my jaw, [it] quite affected me. (patient 82)” “It sounds a bit odd but it would be nice to blend into the background a bit more. (patient 100)” (p. 118)  “When I was told about it at the clinic I started noticing how far back my jaw was. (patient 65)” “Didn’t notice the problem with my bite [until I went to clinic] because I am used to it. (patient 66)” “I am doing it more [not letting people look at the profile of my face] since the clinic it made me worse, more aware. (patient 103)” (p. 118) | Unequivocal |
| Was the information provision sufficient to enable informed choice? | “When you are at the clinic you forget to ask important information but it is all explained in the leaflets. (patient 8)” “The literature helped me to find questions which I could then ask about. (patient 52)” “Need things simplified so that I can understand ... [they] talk in medical terms. (patient 75)” “I did not know about the operation until about a year into treatment. (patient 35)” “The stuff I got before the operation was more useful than the stuff I got originally. (patient 15)” “If I was told at the start that it would of have been two years, I would have been mentally prepared for it … (patient 73)” “The information was more about what it would look like after but not about the process of treatment. (patient 28).” (pp. 118-119)  “I tried not to think about the bad things they said ... I just wanted it [surgery] done. (patient 12)” “I didn’t want to know the ins and outs [of surgery] I just wanted to do it. (patient 28)” (p. 119) | Unequivocal |
| Did patients make reasoned choices about the treatment options? | “I’m prepared to go through the pain if it makes me feel better about myself. (patient 75)” “Always thinking about it … playing it over in my mind. (patient 14)” “Feel I made the right decision for where I am in life. (patient 10)” (p. 119)  “Now I look normal. That’s quite important as it is not cosmetic surgery. As the aim was not to look beautiful just normal. (patient 84)” “I think it increased my self-esteem, not that I realized before the operation that I was conscious of my appearance. (patient 94)” (p. 119)  “Worried that I will look better after surgery but I’ll still feel the same. (patient 100)” “I may come out looking worse than I did before or just the same, I’d go through all that pain and just look as peculiar. (patient 100)” (p. 119)  “They didn’t put it across as a risk but when putting the break in the jaw to move it about, it may split into more pieces. (patient 48)” “General anaesthetics [that’s] dodgy. (patient 1)” (p. 119)  “I didn’t feel I was given an option … (patient 3)” “Long-winded process because once started hard to stop. (patient 12)” (p. 120)  “Yes [risk of no treatment], to my sanity. It would have got me down eventually. (patient 58)” “It could cause premature ageing. (patient 65)” (p. 120)  “They said if I took the brace off it would go back to before. (patient 18)” “They said it would make my nose look huge as my bottom jaw is too small. (patient 8)” (p. 120) | Unequivocal |
| Who influenced the choices about orthognathic treatment? | “As a child, it was my parents’ decision, I had to accept it. (patient 84)” “My dentist said he always regretted not having treatment when he was younger, this influenced me to go from 90% sure to 100% sure to have treatment. (patient 51)” “Did it for me and for my children so they are not embarrassed in the future by me. (patient 84)” (p. 120) | Unequivocal |
| What affect (emotion) was associated with patients’ orthognathic treatment choices? | “Difficult [decision] as neither consultant gave a definite opinion whether I should have it. (patient 90)” “I don’t know. I thought it was my own fault for not wearing the first brace and I thought it was my own fault because of brace … (patient 69)” “About a week before the operation it had to be cancelled [due to abscess] and it was put back a year. I was devastated and almost dropped out but I decided to go on. (patient 28)” (p. 120) | Unequivocal |

**Paul NR, Baker SR, Gibson BJ. Decision making from the experience of orthognathic surgery patients: a grounded theory approach. JDR Clin Trans Res. 2022;7(3):256-66.**

| Findings | Illustration from Publication (page number) | Evidence |
| --- | --- | --- |
| Central role of the dental care professionals in patients’ experience of decision making for orthognathic treatment | “I just put my trust in them [orthodontist and oral surgeon] … you know they are the expert. (Lola, 21 y, female, 2 y postsurgery)” “They are the expert so I have listened to everything they have said. (Millie, 17 y, female, 6 wk postsurgery)” “My dentist, not within the [name of hospital], my own dentist, she is quite pushy, quite fiery and she was quite pushy about saying well why, why don’t you get it done again, not in a negative way but yeah she seemed quite enthusiastic about me going back and getting it sorted. (Emma, 26 y, female, 8 wk postsurgery)” “I don’t exactly have an underbite … my teeth meet exactly. Looking at my profile, I could benefit from orthognathic surgery (the very suggestion made me put off visiting another orthodontist for 18 years!). But I’m not going with the surgery. … The jaw change from surgery would really be very minimal, and I don’t really want an Extreme Makeover. Has anyone else decided ‘no, thanks . … surgery’s not for me and I’ll just go with the straight teeth, thanks!’ (Valster, online: http://www.archwired.com/phpbb2/viewtopic.php?t=15100)” (p. 260) | Unequivocal |
| Awareness and decision making in orthognathic treatment | “Right it was pointed out to me by a friend who like taking a mickey sometimes at me … it was in a joke way but you could tell that there was something wrong with your teeth. So I did [orthognathic surgery] it for myself, like to do the operation and sort it out! … Yeah yeah the teasing triggered the decision probably. If it wouldn’t I would have had it myself. I would have known it a bit later that I need straighter teeth and get better looking. (Kenny, 18 y, male, 6 wk postsurgery)” (p. 261)  “I found it quite umm clear … umm everything was umm well explained, it was very clear what my choices were and it was left to me to make that decision. (April, 19 y, female, 8 wk postsurgery)” “No … no when they mentioned that the jaw was out and that I needed some form of surgery that was when I noticed it. … They said something about the bite which was a bit daunting because I was there thinking of having a brace, maybe have it for two years. But surgery. … (April, 19 y, female, 8 wk postsurgery)” (p. 261) | Unequivocal |
| Information and decision making | “I guess I was expecting more comprehensive support throughout this process. So far it seems like I have to ask all the questions, and that no information is volunteered or presented in any way other than verbally. I'm such a novice in this process, and it's been exhausting to ask question after question and have to dig for information. My mindset alone has shifted on several points since getting serious about braces just over a month ago. (Gabriella36, 33 y, female, online: <http://www.archwired.com/phpbb2/viewtopic.php?t=48706>)” “I felt the less I knew about things the better. … Because I think if you overload yourself with information you are always gonna talk yourself out of it. (Lola, 21 y, female, 2 y postsurgery)” “I found it quite clear, everything was umm well explained, it was very clear what my choices were umm and it was left to me to make that decision coz obviously it’s me that has to go, had to go through the treatment. Everyone I spoke to were very clear, yeah I think I found that (decision making) process the simpler part as it were. (Mia, 28 y, female, 8 wk postsurgery)” “I think it’s one of the most useful things being able to talk to people who have gone through it. I think that was what I found most useful. I had one of my friends who had had the surgery, I spoke to her and also I spoke to a lot of people through the blog I wrote online. (Roxy, 22 y, female, 1 y postsurgery)” (pp. 261-262) | Unequivocal |
| Temporality | “I think it was … I had my children and I think there was quite a bit of pain side of it (laughs) got used to the pain side of it, not thinking of any more children now so sorting myself out … in a way. (Suzie, female 38 y, 6 wk postsurgery)” “I think my difficulties in making the decision were really the extent of the treatment needed, the length of time taken and the impact it had on my life especially since I have only a couple of years left in university and then treatment would still be ongoing when I am trying to find a job on the graduate scheme and into obviously that process. (Will, 21 y, male, decisionmaking phase)” (p. 262) | Unequivocal |
| Motivation and expectations | “Umm … just to look normal. To … have a normal smile and a nice smile … a normal chin, a normal bottom jaw … normal as anyone. (Marie, 66 y, female, 8 wk postsurgery)” (p. 262)  “Leading up to the surgery I was still having speech difficulties, I could speak clearly, annunciating was a problem, I was seeing a speech therapist so I was expecting that would improve. Confidence was the main one really, I just didn’t … I would shy away from myself and just didn’t want to draw attention to myself. (Becky, 22 y, female, 2 y postsurgery)” (p. 262) | Unequivocal |
| Social support | “Each dentist that I have been to, I have mentioned it but they said it’s extreme to go for the surgery so … more or less put me off it and then I moved to [name of town] and then the dentist at [name of another town] suggested it coz he had got people that had it done, sent before for it and it was something he would recommend. So … basically swapping dentists made me have it [orthognathic surgery] done. (Suzie, 38 y, female, 8 wk postsurgery)” (p. 263) | Unequivocal |
| Patient fears—hospitalization and negative results from surgery | “Umm having a stay in the hospital is obviously a big negative. … Also having the surgery itself in the first place is scary. I have never had surgery before so that also comes in. (Will, 21 y, male, decision-making phase)” “I knew about the weight loss … how much will I lose … because I wasn’t a big person anyway. Umm even though my husband was doing very high-calorie foods it wasn’t so much how much weight I lost, I probably lost ¾ of a stone but it was the body mass I lost, my clothes were out here (showing action) and umm for about 3-4 months I was like that, you really don’t realise how much weight loss you do with that. That was one of my fears. (Kathy, 52 y, female, 2 y postsurgery)” (p. 263) | Unequivocal |

**Broder HL, Phillips C, Kaminetzky S. Issues in decision making: should I have orthognathic surgery? Semin Orthod. 2000;6(4):249-58.**

| Findings | Illustration from Publication (page number) | Evidence |
| --- | --- | --- |
| Health attitudes | “This (the treatment) is like an investment in myself.” “My fantasy would be to go back to Costa Rica and see all those guys that used to make fun of me. I would just tell them who I am... When this is over I will do whatever I want in public: talk, smile, whatever. There are no limits.” “After treatment is over I might meet someone and could have a lot of fun. I was the odd man out all the time.” “Eating a steak and carrots, speaking more clear so everyone understands me.” (pp. 253-254)  Concern about the consequences of surgery, ie, temporary and/or permanent sensory disturbances, swelling and pain, and extent of facial change (“too much” or “why bother”). (p. 255) | Unequivocal |
| Family stress | Among the older patients, now having grown children, time for myself, money saved, insurance, transportation, and a regular job were among some of the intrapersonal factors facilitating the health-seeking behaviors. (p. 254) | Unequivocal |
| Logistics/quality of care | Complaints about the waiting time, the length between appointments, securing financial coverage and treatment plans were discussed. (p. 255) | Unequivocal |
| Facility/physical environment | “Personally I am really picky, I like everything clean. Sometimes I come from work and really need to go to the bathroom, but the bathroom has nothing to do with the chair the doctor works in but if is a dirty bathroom, then I am thinking they do not really keep this place clean.” “I like it simple, the bells and whistles don’t spin me.” “I like the cost-I accept that a school may have older stuff, but the price is real good.” (p. 255) | Unequivocal |
| Interpersonal communication | Most of participants said that it was their decision but conferred with others to legitimize their concerns and endorse their treatment-seeking behaviors. Each of the orthognathic patients had either known someone who had had such care or was favorably impressed with the doctor-patient communication about treatment. (p. 254)  “My doctor is terrific, really knows what he is doing.” “I would not go to that guy, it was like a salesman’s pitch and I didn't think he was concerned about me.” “I kind of formed a bond with her, she was really sweet and I looked forward to meeting with her every month.” “being taken seriously” and not “being talked down to.” (pp. 254-255) | Unequivocal |
| Access to care | “I have wanted to fix my teeth forever, but my mother could not afford it when I was young.” “I never knew that adults could get braces.” “I never knew I looked so bad. I didn't know that everyone saw me looking that way.” “I really like the 3-D stuff-it showed me how I would look and I liked how I was gonna look.” (p. 254) | Unequivocal |

**Ansari UH, McNeilage AG, Ashton-James CE, Campbell J, Wong E, Lim L. Perioperative patient perspectives on orthognathic surgery - a prospective study. Ann Maxillofac Surg. 2024;14(1):46-51.**

| Findings | Illustration from Publication (page number) | Evidence |
| --- | --- | --- |
| Hope for aesthetic improvements | “My mouth has got an overbite”. P09  “My double chin will go”. P12  “The little kink in my nose gets straightened up”. P03  “I have a flat face profile”. P05  “My teeth are always bucky”. P09  “It’s a bit gummy”. P14  “There’s some research that suggests that having proper facial structure helps with that, sort of circulations and things”. P08 (p. 49) | Credible |
| Hope for socio-emotional improvements | “Repulsed by my own reflection”. P14  “Really insecure about my face”. P17  “It’s been making me feel down and it’s really affected my self‑esteem and my confidence”. P11  “Excited to actually smile in photos, post on Instagram with me showing my teeth and everything…”. P13  “More comfortable in my own skin”. P11  “Get back out into the world”. P04 (p. 49) | Unequivocal |
| Hope for functional improvements | “I get to speak properly, breathe properly, eat properly, be a healthier person”. P08  “Some people, they don’t understand what I’m saying”. P06  “Sometimes my voice can sound a bit muffled”. P10 (p. 49) | Unequivocal |
| Hope for reduced pain | “I’ll be able to eat food and not worry about my jaw hurting”. P15  “I can’t really yawn normally. because it just hurts that much”. P17 (p. 49) | Unequivocal |
| Fear of general surgical risks | “With every surgery, you have to feel concerned”. P06  “There’s always a slight chance of surgery not going well”. P08  “…what if I wake up and feel pain, always the worst‑case scenario”. P15  “It’s just sort of some irrational nerves basically”. P08  “I have a slightly higher chance of bleeding out”. P08  “I’ve got a lot of allergies, so what if I’m allergic to any medication …?”. P11 (p. 49) | Unequivocal |
| Fear of specific surgical risks | “There’s a lot of nerves, it might just clip, or something”. P09  “Having to go back in”. P11  “I know my doctor’s got a lot of experience and I feel confident”. P11  “If they didn’t know what they were doing, they wouldn’t be in that room”. P04 (p. 49) | Credible |
| Fears with post-surgery recovery | “Put life on hold”. P12  “Because it stopped me from doing a few things which I won’t be able to do, which is very sad”. P04  “That’s when I become like… a bit depressed”. P12 (p. 49) | Unequivocal |
| Fear of changing appearance | “You spend twenty years looking into the mirror and seeing the same face and then… one minute, its changing”. P04  “I’m hoping I can still look in a mirror and recognise myself”. P04  “I’m just nervous of what people will think after it”. P13 (p. 49) | Unequivocal |
| Anxiety and excitement | “I’m more excited than scared to be honest”. P06  “I’m slightly nervous about the operation itself, but overall, I’m quite optimistic”. P08  “I am a bit terrified, but I’m also eager to do it”. P12  “Obviously very nervous, but excited as well”. P13  “I am a little bit concerned. but I think my excitement overtakes it”. P11 (p. 49) | Unequivocal |
| Being told that they needed it | “…I didn’t know nothing about this surgery until my orthodontist told me, gave me the rundown”. P09  “Now that my doctor and I have spoken about it, and I know that it’s not normal, I’ve been noticing it a lot and it’s been making me feel down...”. P11 (p. 49) | Unequivocal |
| Social stigma | “I think there’s some controversy of people nowadays getting surgery and like some people just don’t understand that it’s not always wanting to be someone else or looking a certain way, it’s like for your health as well”. P08 (p. 49) | Unequivocal |

**Antov H. Investigating patient and clinician perceptions of facial profiles and the psychological aspects of orthognathic care. Doctor of Dental Science. University of Liverpool; 2023.**

| Findings | Illustration from Publication (page number) | Evidence |
| --- | --- | --- |
| Psychosocial effects | “Oh god I hate it. It’s just… I’m like a bulldog, just all chin. It’s all I can see when I look at myself. I hate it.” (Patient Int 14, pg 6, line 182)  “Yeah, I'd say anxiety really. I've been diagnosed with anxiety by my GP and I think a lot of it stems from my appearance and feeling abnormal. Being bullied at school, not feeling good enough. All that kind of stuff, really.” (Patient Int 9, pg 4, line 123)  “So, the way I do make up, I always make sure I have defined cheekbones, and things like that, and I put my head down in pictures. So definitely anxiety, I’d never get a picture taken, just stuff like that.” (Patient Int 12, pg 5, line 163)  “Any big gatherings I don’t do well in. It just feels like I stand out and everyone looks at me. So, it does stop me, I don’t… Often I don’t go if there will be many people.” (Patient Int 10, pg 5, line 197)  “It’s difficult because I’m not as confident as I could be you know.” (Patient Int 7, Pg 3, Line 96)  “So, you have to go either way. You either retreat and become this quiet person so nobody will notice you. Or you do what I did and go the opposite way, and just become really confident and outgoing. To try and distract from it.” (Patient Int 12, pg 7, line 231)  “Yeah, I was bullied at school. And of course, the focus was always my jaws, they would say things like its goofy and so on.” (Patient Int 7, pg 8, line 220)  “It makes you much more self-conscious you know… I feel like it did make it a much bigger part of my life. Before that I had noticed my jaws, but after kids started bullying me, I became almost obsessed by them.” (Patient Int 10, pg 9, line 281) | Unequivocal |
| Researching orthognathic surgery | “I just wasn’t sure what was wrong with me exactly, I knew something was off, and so when my dentist saw me and mentioned this option…I was very keen to see the specialists. I wanted their expert opinion, you know.” (Patient Int 8, pg 3, line 98)  “They really went into detail analysing my jaws from all angles. It was great, because they are experts…you know. It… I knew what I disliked about them, but they managed to explain it in much clearer detail, and explained exactly where the issues were.” (Patient Int 2, pg 6, line 221)  “It’s hard to just have one appointment with you guys and you tell us about it and give a leaflet, but the leaflet I’ll be honest is so short and simple, with little drawings, I don’t think it gives enough information.” (Patient Int 7, line 137, pg 5)  “And I follow many people having the procedure, from Russia, from Singapore, USA, England many places. It’s amazing, and you see them go through it a few steps ahead.” (Patient Int 7, pg 4, Line 135)  “I’m not sure about specifics, I don’t remember. I know there will be pain afterwards.” (Patient Int 3, pg 6, line 281)  “There are many risks, and I’ve done my research, I’ve seen the possible consequences. There is death, blindness, loss of muscle movement, nerve damage, infections. You can get facial changes you don’t want, so it’s not fully predictable.” (Patient Int 2, Pg 6, line 52)  “It was great to finally have someone acknowledge it you know, confirm that I’m not crazy. But it does make it more real. You know when you have two doctors saying you should have major surgery, it kind of makes it more of a thing if that makes sense.” (Patient Int 3, pg 5, line 161) | Unequivocal |
| Self-perception and agreement with clinicians | “The chin makes me look like a witch! It’s very pointy, and it’s all you can see. It’s wonky and just terrible.” (Patient Int 9, pg 4, line 112).  “It’s massively affected my self-esteem. I don’t feel confident, especially in social situations. But even you know, when I have an interview, I dread it, I’m worried about them looking at my jaw.” (Patient Int 6, pg 5, line 146)  “I really liked them; they knew exactly what bothered me. Straight away we agreed.” (Patient Int 3, pg 4, line 133)  “Kind of. But I don't think they see it as I do. I think they see me as a clinical case. I like them. But I felt like it wasn’t like, “oh she is feeling this sort of way”. That empathy is not there. They discuss, clinically this is what it looks like, so clinically we can do this and that etc.” (Patient Int 12, Pg 8, line 271) | Credible |
| Expectation and motivation | “I’d expect to you know, still look like myself, but just with a less prominent jaw, the bite would not be reversed.” (Patient Int 13, pg 7, line 244)  “My cheekbones would fill out, and my nose would look more balanced, less obvious. Right now it dominates my face. So, the face would just become more balanced you know.” (Patient int 6, Pg 3, Line 108)  “I think it would make me a bit more confident.” (Patient Int 11, pg 7, line 228)  “I knew straight away it would change my life.” (Patient Int 7. Pg 2. Line 48)  “I think people would take me more seriously.” (Patient Int 11, Pg 2, line 37)  “Idea of taking pictures and the jaws looking nice really encourages me.” (Patient Int 7, Pg 4, line 108)  “My family are very encouraging, especially my sister who has had it.” (Patient Int 5, Pg 5, Line 124)  “My family and girlfriend have been massively supportive.” (Patient Int 7, Pg 4. Line 126)  “And my mum, she thinks I’m absolutely bonkers. She thinks I’m crazy. She went with my sister to her consultation, and they never consented her at the time because my mum was just like “no, no, no”. So, she had to go home and come back.” (Patient Int 12, pg 4, line 135)  “Well, you do notice people starring. And for example, one of my colleagues brought her little girl into work, and I swear to you Hans this little girl would not stop starring at my jaw *laughs*, it was terrible, I just wanted to shrink.” (Patient Int 11, pg 5, line 147) | Unequivocal |
| Service experience and improvement | “I think it’s a great idea. Its someone else to talk to, a professional who would understand, who would know what to say. I mean no offence to you guys, but I wouldn’t exactly tell XXXX (patient’s OMF surgeon) or XXXX (patient’s orthodontist) about my mental health you know, it’s not their job.” (Patient Int 4, pg 5, line 161)  “It’s a bit like following the people on social media. I think it would make a massive difference to have someone go through it with you, or before you even so you can ask them questions.” (Patient Int 7, pg 6, line 179)  “Long waiting period but overall smooth journey.” (Patient Int 7, Pg 1, Line 28)  “It was a wonderful feeling, imagine you have a condition, and you think nothing can be done with it, you have to live with it your whole life, then you suddenly discover there is an option. It was such relief.” (Patient Int 6, pg 3, line 103)  “I know I’m in good hands.” (Patient Int 7, Pg 4 Line 119) | Unequivocal |

**Travess HC, Newton JT, Sandy JR, Williams AC. The development of a patient-centered measure of the process and outcome of combined orthodontic and orthognathic treatment. J Orthod. 2004;31(3):220-34.**

| Findings | Illustration from Publication (page number) | Evidence |
| --- | --- | --- |
| Facial appearance | “That was why I did it, the only reason I did it. Because I have never ever liked the way I looked.” (p. 227) | Unequivocal |
| Difficulty eating | “I always choose, or I always chose things that were easy to eat. I would never, ever have a piece of meat, like a steak, which involved a lot of chewing.” (p. 227) | Unequivocal |

**Bhamrah G, Ahmad S, NiMhurchadha S. Internet discussion forums, an information and support resource for orthognathic patients. Am J Orthod Dentofac Orthop. 2015;147(1):89-96.**

| Findings | Illustration from Publication (page number) | Evidence |
| --- | --- | --- |
| Difficulties with function | “I was sick and tired of not being able to enjoy food properly.”  “I'm constantly pushing my lower jaw forward in order to speak properly without my lower lip being caught under my upper teeth.”  “I have a problem with my airway, makes it difficult to breathe at night.” (pp. 90-91) | Unequivocal |
| Unhappiness with appearance | “I hardly ever show my teeth whilst smiling, can't wait for the day I feel confident enough to smile properly.”  “I became really insecure about my profile as my lower jaw just kept growing longer and people kept pointing it out.” (p. 91) | Unequivocal |

**Watts GD, Christou P, Antonarakis GS. Experiences of individuals concerning combined orthodontic and orthognathic surgical treatment: a qualitative Twitter analysis. Med Princ Pract. 2018;27(3):227-35.**

| Findings | Illustration from Publication (page number) | Evidence |
| --- | --- | --- |
| Surgical procedure | “I need this today. I’m trying to be fearless in what is coming my way but I’m still scared #JawSurgery”  “Getting mentally prepared for jaw surgery! #Nervous #Excited #JawSurgery” (p. 5) | Credible |
| Outcome | “T minus one day. I’ll be at the hospital on exactly 24 hours! Almost one step closer to eating a sandwich that won’t fall apart #jawsurgery”  “I can’t wait to get jaw surgery & finally have perfect teeth I don’t even care about the recovery I could get it tomorrow” (p. 5) | Unequivocal |
| Financial issues | “I begged my mom to pay for my jaw surgery when I got braces but she said no and it’s my biggest insecurity”  “Do you not understand I’m poor especially if I get the jaw surgery I can’t buy no Macbook Pro” (p. 5) | Unequivocal |

**Kettle JE, Marshman Z, Winchester L, Hardwick L, Bolt R, Lee N. Patients’ narratives of orthognathic treatment for facial asymmetry: a qualitative study. Br J Oral Maxillofac Surg. 2021;59(4):445-53.**

| Findings | Illustration from Publication (page number) | Evidence |
| --- | --- | --- |
| Becoming aware | “One side of my jaw had carried on growing and the other one hadn’t. I was really aware of the shape of my chin or the shape of my jaw. I hated that when I smiled, you could see lots of gum.” (P13)  “When I got referred to the orthodontist to look at my teeth, that’s when they said, you’ve got all these problems with your jaw and your face is asymmetric.” (P1)  “They used to tell me that I just looked the same as everyone else but I didn’t.” (P2) (p. 449) | Unequivocal |
| Negative impacts of asymmetry | “I didn’t like going out. My friends used to invite me out and because obviously I felt not as good as they did, so I used to not go.” (P12)  “I could actually show you the whole picture, there’s about six of us in this picture here. They all look, right nice, so then it’s just me that constantly sticks out.” (P4)  “I couldn’t eat very well. Having to eat in front of people and not do it as good as them, it was just a lot of teasing, a lot of nastiness.” (P14)  “It was just very uncomfortable. I was getting headaches constantly, aching jaws, and I just didn’t feel right.” (P7)  “It was just kind of, people are noticing this, and obviously that made me depressed.” (P6) (p. 449) | Unequivocal |
| Committing to treatment | “I’d already decided 10-15 years ago, whatever it involved doing, I want to take it as far as possible and see where we can go with it. Once everyone said, ‘Yes, we can do something and this is what we’re going to do,’ then my mind was never going to falter from whatever it was that I needed to do.” (P6)  “My mum was really concerned, even[though] like I left home 20 years ago she was obviously, they’re all like, ‘You don’t need to get it done.’” (P7) (p. 449) | Unequivocal |
| Establishing expectations | “I think everyone was really clear about what was going to happen and how it was going to progress. They told me from the start, it’s going to be a three-year kind of progress.” (P10)  “I was trying to find someone who had recorded it just to see.” (P8) (p. 449) | Unequivocal |

**Cadogan J, Bennun I. Face value: an exploration of the psychological impact of orthognathic surgery. Br J Oral Maxillofac Surg. 2011;49(5):376-80.**

| Findings | Illustration from Publication (page number) | Evidence |
| --- | --- | --- |
| Self-awareness of appearance | “Before (operation) I felt that I was a lot different, that I was the ugly duckling as it were sort of, in the class.”  “Probably in certain situations I felt a bit insecure, like if you’ve got like a side-view or something, it’s like noticeable.” (p. 377) | Unequivocal |
| Attitudes of others and appearance | “My body image at the time was very low; I pretty much struggled to find anything I liked about myself, which I think simply spread from the sort of unspecific name calling I used to get.”  “You see all these people in the magazines and all that…like all the models and stuff, and all the celebrities and stuff like that, and yeah, I wanted to look like that really.” (p. 377) | Unequivocal |
| Treatment issues | “I suppose it wasn’t normal was it – sticks out a bit from everyone else, so I thought if you could get it improved I may as well do it.”  “I was getting to about 16, 17 – that was when it was getting more noticeable.”  “I did notice it a bit before but I didn’t worry about it as much as when I was 17 or 18.” (p. 377) | Unequivocal |
| Support and coping | “I’m very close to my dad; I tell him everything, um, and my dad has also got a cleft palate so that helped.”  “Different isn’t necessarily ugly. If you don’t think I look any different why do you think other people think you look different?”  “Especially with the help of my friends and the support of my friends and teachers and family afterwards, it does give you a chance to build up more confidence in yourself.”  “My orthodontist, surgeon, and I did have quite a lot of talks about whether I should go through with it and what would be the outcome of it.”  “I still do get called the odd name by the odd person, but the difference with me now is I’ve built up enough confidence and strength in myself to say, ‘that’s your opinion, if you think that, fine, but I don’t care to listen to it’.” (p. 378) | Unequivocal |

**Liddle MJ, Baker SR, Smith KG, Thompson AR. Young adults’ experience of appearance-altering orthognathic surgery: a longitudinal interpretative phenomenologic analysis. Cleft Palate Craniofac J. 2018;55(2):238-47.**

| Findings | Illustration from Publication (page number) | Evidence |
| --- | --- | --- |
| The course of treatment and appearance sensitivity | “I won’t let people take pictures of me or if I do I won’t smile or open my mouth and I don’t really eat out in public as much as I used to.” (Charlotte)  “I think I’ve noticed it a lot more, since it’s been pointed out and that. I think I’m more self-conscious of it as well.” (Joe)  “It’s the little defects what they point out to you, you know you’re sat in that chair and they’re like ‘this and this’ and you’re like ‘oh.’ They said my top lip is a bit flat which I’d never ever noticed it before, and then it was pointed out and then I started  noticing it.” (Chloe) (p. 241) | Unequivocal |
| Uncertainty and never being prepared | “One of my main worries is, just if I’m unhappy with how I look afterwards. I’m, if I don’t like it because it’s like I’ve got to live with it for the rest of my life.” (Emily)  “I just thought I’ve got to have it done. I just had to convince myself that I didn’t really have a choice in it. Because I’d regret it if I would’ve said no, I know I would have.” (Chloe)  “It’s not debilitating in any way but I do think I can’t underplay it because the point that I’m at now is, because I feel as though it’s something that does need treatment.” (John)  “I’d noticed it but it wasn’t a problem for me, but I’ve come for this treatment and I’m looking forward to the end results now.” (Joe)  “I didn’t prepare myself I don’t think for it. … I don’t think I could have prepared myself more, I just, I pushed it to the back of my mind that I were having it done until the day I were having it done, because I get scared of stuff like that so I should have thought about it.” (Fiona)  “I feel like I’ve had to find things out for myself. So … like, up until about five month ago, I only just found out it were gonna change me nose as well. … I didn’t know nowt about that coming through all the years and the process with it.” (Emily) (p. 242) | Credible |
| Impact of actual negative reactions of others | “It were me lad friends who used to say things but then still, they still stick in your mind now. Even though it were when I were at school I still think about it at times.” (Emily)  “I just wanted to fit in. I mean it doesn’t bother me what people think but it’s just, it’s nice to be normal isn’t it?” (Fiona)  “Signs of nervousness or being unsure about anything in particular, regardless of what the reason for that is, erm, can be, er, it can cause people to have a lack of confidence in your ability.” (John) (p. 242) | Unequivocal |
| Influences of significant others on decision making | “Dad says if I had the chance to have it done on the NHS, he say, I’d have it done.” (Chloe)  “It was mainly my mum that pushed me into it to be honest.” (Joe)  “From seeing a lot of before and after pictures that they’ve shown me already it does massively improve your appearance. … There’s not any sort of, that don’t look as good as they did before.” (Charlotte)  “Most of my friends said ‘oh, you don’t need it.’ Most of my relatives said you don’t need it. Apart from my mum again, who said I do need it. … In the end though, if there is a chance that it’s going to get worse it’s better to get it sorted now. So in the end they all agreed, sort of, that I’d need it.” (Joe)  “Everyone’s supported me and they’d all just think that it’s my decision, whatever I decide I want to do.” (Emily)  “It was having my family around me that, you know, helped me through the day.” (Joe)  “Everybody involved with the treatment has been absolutely fantastic.” (John)  “I think people who work at [dental hospital] who I’ve been seeing on a regular basis who are very helpful and supportive.” (Emily) (pp. 242-243) | Unequivocal |

**Appendix C: Findings, categories & synthesized findings**

| Synthesized findings | Categories | Findings |
| --- | --- | --- |
| Synthesized finding 1:  Initial intention: Inner drivers and self-reconstruction | Functional and aesthetic needs | Aesthetic needs |
|  |  | Psychological needs |
|  |  | Functional improvement |
|  |  | Nature of the problem |
|  |  | Trigger for treatment now |
|  |  | Source of motivation |
|  |  | Functional or appearance expectations |
|  |  | Motives for seeking orthognathic treatment |
|  |  | Did patients make reasoned choices about the treatment options? |
|  |  | Awareness and decision making in orthognathic treatment |
|  |  | Motivation and expectations |
|  |  | Health attitudes |
|  |  | Hope for aesthetic improvements |
|  |  | Hope for functional improvements |
|  |  | Hope for reduced pain |
|  |  | Social stigma |
|  |  | Psychosocial effects |
|  |  | Self-perception and agreement with clinicians |
|  |  | Expectation and motivation |
|  |  | Facial appearance |
|  |  | Difficulty eating |
|  |  | Difficulties with function |
|  |  | Unhappiness with appearance |
|  |  | Outcome |
|  |  | Becoming aware |
|  |  | Negative impacts of asymmetry |
|  |  | Self-awareness of appearance |
|  |  | Attitudes of others and appearance |
|  |  | Treatment issues |
|  |  | The course of treatment and appearance sensitivity |
|  | Psychosocial needs | Psychological needs |
|  |  | Nature of the problem |
|  |  | Impact on interpersonal relationships |
|  |  | Impact on employment |
|  |  | Impact on confidence |
|  |  | How the problem makes them feel |
|  |  | Reason for wanting treatment |
|  |  | Emotional effects |
|  |  | Social effects |
|  |  | Psychological effects |
|  |  | Lifestyle effects |
|  |  | Motives for seeking orthognathic treatment |
|  |  | Did patients make reasoned choices about the treatment options? |
|  |  | Motivation and expectations |
|  |  | Health attitudes |
|  |  | Hope for socio-emotional improvements |
|  |  | Psychosocial effects |
|  |  | Self-perception and agreement with clinicians |
|  |  | Expectation and motivation |
|  |  | Negative impacts of asymmetry |
|  |  | Attitudes of others and appearance |
|  |  | Impact of actual negative reactions of others |
|  | Self-schema | Coping strategies |
|  |  | Metamorphosizers |
|  |  | Pragmatists |
|  |  | Shedders |
|  |  | Evolvers |
|  |  | Did patients make reasoned choices about the treatment options? |
|  |  | Psychosocial effects |
|  |  | Support and coping |
|  | Alternative treatments | Aesthetic needs |
|  |  | Did patients make reasoned choices about the treatment options? |
|  |  | Uncertainty and never being prepared |
| Synthesized finding 2:  Evaluating pathways: Ideal visions versus practical constraints | Surgical outcomes | Surgical outcomes |
|  |  | Did patients make reasoned choices about the treatment options? |
|  |  | Health attitudes |
|  |  | Fear of specific surgical risks |
|  |  | Fear of changing appearance |
|  |  | Uncertainty and never being prepared |
|  | Fear of treatment | Surgical safety |
|  |  | Surgical outcomes |
|  |  | Did patients make reasoned choices about the treatment options? |
|  |  | Patient fears—hospitalization and negative results from surgery |
|  |  | Health attitudes |
|  |  | Fear of general surgical risks |
|  |  | Fear of specific surgical risks |
|  |  | Researching orthognathic surgery |
|  | Treatment duration | Did patients make reasoned choices about the treatment options? |
|  |  | Temporality |
|  |  | Family stress |
|  |  | Logistics/quality of care |
|  |  | Fears with post-surgery recovery |
|  |  | Service experience and improvement |
|  | Financial considerations | Financial situation |
|  |  | Family support |
|  |  | Logistics/quality of care |
|  |  | Facility/physical environment |
|  |  | Access to care |
|  |  | Financial issues |
|  | Service experience | Facility/physical environment |
|  |  | Service experience and improvement |
| Synthesized finding 3:  Confirming pathways: Role of social networks | Information support | Information services |
|  |  | Surgical outcomes |
|  |  | Initial awareness of problem |
|  |  | Trigger for treatment now |
|  |  | Motives for seeking orthognathic treatment |
|  |  | Was the information provision sufficient to enable informed choice? |
|  |  | What affect (emotion) was associated with patients’ orthognathic treatment choices? |
|  |  | Awareness and decision making in orthognathic treatment |
|  |  | Information and decision making |
|  |  | Logistics/quality of care |
|  |  | Access to care |
|  |  | Being told that they needed it |
|  |  | Researching orthognathic surgery |
|  |  | Becoming aware |
|  |  | Establishing expectations |
|  |  | The course of treatment and appearance sensitivity |
|  |  | Uncertainty and never being prepared |
|  |  | Influences of significant others on decision making |
|  | Social influence | Family support |
|  |  | Who influenced the choices about orthognathic treatment? |
|  |  | Interpersonal communication |
|  |  | Expectation and motivation |
|  |  | Service experience and improvement |
|  |  | Committing to treatment |
|  |  | Support and coping |
|  |  | Influences of significant others on decision making |
|  | Role of clinicians | Who influenced the choices about orthognathic treatment? |
|  |  | Central role of the dental care professionals in patients’ experience of decision making for orthognathic treatment |
|  |  | Social support |
|  |  | Interpersonal communication |
|  |  | Fear of specific surgical risks |
|  |  | Researching orthognathic surgery |
|  |  | Self-perception and agreement with clinicians |
|  |  | Service experience and improvement |
|  |  | Support and coping |
|  |  | Influences of significant others on decision making |
| Synthesized finding 4:  Decision timing: Why now? | Trigger event | Trigger for treatment now |
|  |  | What affect (emotion) was associated with patients’ orthognathic treatment choices? |
|  |  | Awareness and decision making in orthognathic treatment |
|  | Psychology before surgery | Did patients make reasoned choices about the treatment options? |
|  |  | What affect (emotion) was associated with patients’ orthognathic treatment choices? |
|  |  | Anxiety and excitement |
|  |  | Service experience and improvement |
|  |  | Surgical procedure |
|  |  | Uncertainty and never being prepared |

**Appendix D: Summary of robustness checks**

| Condition | Synthesized findings (n=4) | Categories (n=14) | Details |
| --- | --- | --- | --- |
| Excluding studies exclusively using social media data [44, 45] | All 4 retained | All 14 retained |  |
| Excluding a dissertation [32] | All 4 retained | All 14 retained |  |
| Excluding studies with the lowest-quality JBI scores [29, 42] | All 4 retained | All 14 retained |  |
| Excluding studies focusing on surgical experience [46-48] | All 4 retained | All 14 retained |  |

**Appendix E: Summary of cross-contextual comparisons**

| Condition | Synthesized findings (n=4) | Categories (n=14) | Details |
| --- | --- | --- | --- |
| By healthcare system | | | |
| NHS group [30-32, 39, 40, 43, 46-48] | All 4 retained | 13/14 retained | Category 8 (financial considerations) absent |
| Non-NHS group [29, 41, 42, 44, 45] | All 4 retained | 12/14 retained | Category 3 (self-schema) and category 13 (trigger event) absent |
| By treatment outcome | | | |
| Surgery-only group [30, 32, 39, 42, 43, 46-48] | All 4 retained | 13/14 retained | Category 8 (financial considerations) absent |
| Mixed group [29, 31, 40, 41, 44, 45] | All 4 retained | All 14 retained |  |
